# Ionizable drug delivery systems for efficient and selective gene therapy

**DOI:** 10.1186/s40779-023-00445-z

**Published:** 2023-02-27

**Authors:** Yu-Qi Zhang, Ran-Ran Guo, Yong-Hu Chen, Tian-Cheng Li, Wen-Zhen Du, Rong-Wu Xiang, Ji-Bin Guan, Yu-Peng Li, Yuan-Yu Huang, Zhi-Qiang Yu, Yin Cai, Peng Zhang, Gui-Xia Ling

**Affiliations:** 1 Faculty of Medical Device, Shenyang Pharmaceutical University, Shenyang, 110016 Liaoning China; 2 Wuya College of Innovation, Shenyang Pharmaceutical University, Shenyang, 110016 Liaoning China; 3 School of Pharmacy, Yanbian University, Yanji, 133002 Jilin China; 4 School of Pharmacy, Shenyang Pharmaceutical University, Shenyang, 110016 Liaoning China; 5 Masonic Cancer Center, University of Minnesota, Minneapolis, MN 55455 USA; 6 Masonic Cancer Center and Department of Medicinal Chemistry, University of Minnesota, Minneapolis, MN 55455 USA; 7 Advanced Research Institute of Multidisciplinary Science; School of Life Science; School of Medical Technology; Key Laboratory of Molecular Medicine and Biotherapy; Key Laboratory of Medical Molecule Science and Pharmaceutics Engineering, Beijing Institute of Technology, Beijing, 100081 China; 8 Department of Laboratory Medicine, Dongguan Institute of Clinical Cancer Research, Affiliated Dongguan Hospital, Southern Medical University, Dongguan, 523018 Guangdong China; 9 Department of Health Technology and Informatics, The Hong Kong Polytechnic University, Hong Kong, Hong Kong SAR China

**Keywords:** Ionizable nanomaterials, Ionizable drug delivery systems (IDDSs), Nucleic acids, Gene therapy

## Abstract

Gene therapy has shown great potential to treat various diseases by repairing the abnormal gene function. However, a great challenge in bringing the nucleic acid formulations to the market is the safe and effective delivery to the specific tissues and cells. To be excited, the development of ionizable drug delivery systems (IDDSs) has promoted a great breakthrough as evidenced by the approval of the BNT162b2 vaccine for prevention of coronavirus disease 2019 (COVID-19) in 2021. Compared with conventional cationic gene vectors, IDDSs can decrease the toxicity of carriers to cell membranes, and increase cellular uptake and endosomal escape of nucleic acids by their unique pH-responsive structures. Despite the progress, there remain necessary requirements for designing more efficient IDDSs for precise gene therapy. Herein, we systematically classify the IDDSs and summarize the characteristics and advantages of IDDSs in order to explore the underlying design mechanisms. The delivery mechanisms and therapeutic applications of IDDSs are comprehensively reviewed for the delivery of pDNA and four kinds of RNA. In particular, organ selecting considerations and high-throughput screening are highlighted to explore efficiently multifunctional ionizable nanomaterials with superior gene delivery capacity. We anticipate providing references for researchers to rationally design more efficient and accurate targeted gene delivery systems in the future, and indicate ideas for developing next generation gene vectors.

## Background

Gene therapy is a potential therapeutic strategy [1], which has been more and more widely applied in the diagnosis and treatment of various illnesses, including inflammatory diseases, viruses, vaccines, cancer, neurological disorders, and so on [2]. In particular, during the global outbreak of coronavirus disease 2019 (COVID-19), the successful development and application of the mRNA vaccine highlighted the advantages, accelerated the development process, and predicted broad prospects of gene therapy [3].

However, safe and efficient delivery systems, optimized design and preparation process meeting commercial production standards are still the main factors affecting its clinical application and industrial development for a long time [4]. To overcome these difficulties, researchers have been making unremitting efforts. In 2016, scientists found an appropriate solution by developing the chemical modification of nucleic acid monomers as a key technology to solve the problems of stability, off-target effects and immune stimulation effects of nucleic acids [5]. In particular, antisense and RNA interference (RNAi) strategies play a critical role in the study of nucleic acid therapeutics. Short double-stranded RNAs are created by RNAi, which is defined by their loading into the RNA-induced silencing complex. In addition to chemical modification techniques, the successful clinical translation of nucleic acid therapy is also related to the innovation of delivery vehicles. The delivery vehicles can preserve nucleic acids against immunological components and serum nucleases, and also change the drug biodistribution. In 2018 and 2019, the first small nucleic acid interference drug Patisiran and Givosiran were approved by the U.S. Food and Drug Administration (FDA) to treat hereditary transthyretin- mediated (hATTR) amyloidosis [6] and acute hepatic porphyria [7], respectively. To our knowledge, COVID-19 has caused not only a major health medical burden but also an economic crisis since 2019 [8, 9]. Most fortunately, the BNT162b2 vaccine [10–12] has been approved as the first COVID-19 vaccine by FDA and officially authorized with complete phase III experiment in the world [13], which becomes one of the most powerful shields against COVID-19 viruses [14–17].

The most critical contributor to this great breakthrough is the application of ionizable nanocarriers in the transfer of nucleic acids [17–22]. With the emerging development of ionizable nanomaterials, such as ionizable lipids, ionizable phospholipids and ionizable polymer-lipids, the ionizable drug delivery systems (IDDSs) have been extensively investigated and become currently the most sophisticated carriers for the delivery of nucleic acid medications [23]. The common IDDSs include ionizable lipid-mediated drug delivery systems [24, 25], ionizable polymeric nanosystems, and ionizable polymer-lipid nanoparticles (IPLNPs) [26] for the delivery of small interfering RNA (siRNA), messenger RNA (mRNA), small guide RNA (sgRNA), small activating RNA (saRNA), and plasmid DNA (pDNA) [27], among which siRNA and mRNA have been studied the earliest with the most studies and the widest application ranges.

At present, the major mRNA vaccines of COVID-19 are the BioNTech vaccine and the Moderna vaccine [28]. The main active component of BioNTech vaccine is BNT162b2 mRNA with ionizable lipids (ALC-0315), cholesterol, polyethylene glycol (PEG, ALC-0159), and neutral auxiliary phospholipids as auxiliary components. Moderna vaccine is composed of mRNA-1273, ionizable lipids (SM-102), cholesterol, PEG (PEG2000-DMG), and phospholipids [9, 29]. In the IDDSs, the ionizable nanomaterials perform crucial roles. The nucleic acid drugs are successfully encapsulated into IDDSs by complexation with ionizable nanomaterials and swallowed into endosomes [24]. The pH of the nuclear endosome causes disruption of the bilayer structure of IDDSs, releasing nucleic acids that bind to ribosomes responsible for protein production and translation into viral proteins [30].

IDDSs are not only necessary for infectious disease vaccines, but also are emergingly applied in cancer therapy. IDDSs may include nucleic acids that can prevent tumor growth [31]. Compared with other delivery systems, IDDSs can significantly reduce the systemic toxicity of nucleic acid drugs, improve drug delivery efficiency, and enable drugs to achieve better antitumor activity. In addition, it is recognised that destroying gene expression in tumors is a novel approach to treating cancer [32], and targeting nucleic acid treatment has potential applications in non-cancerous tissues and organs [33].

**Fig. 1:**
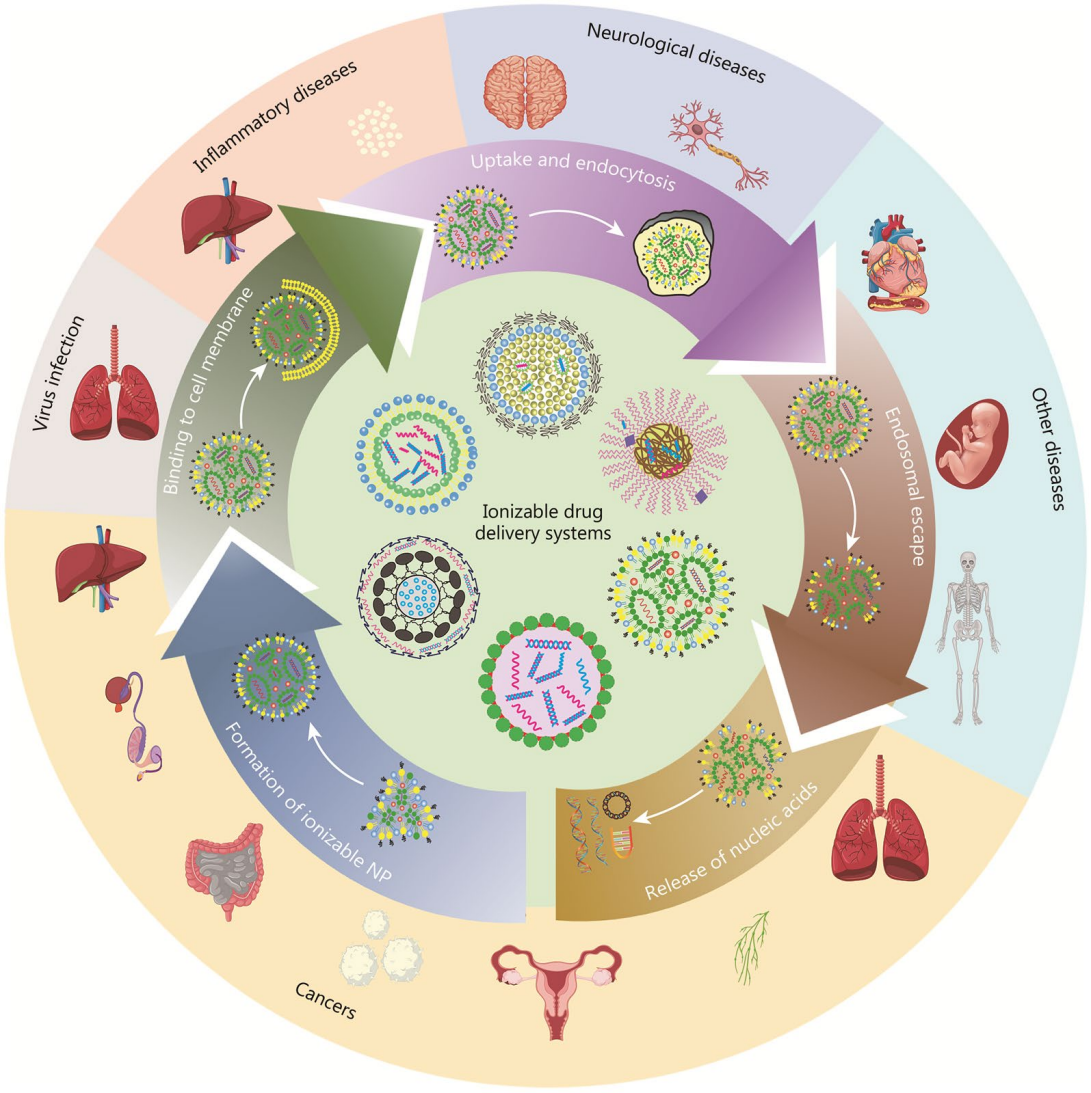
Outline figure of ionizable drug delivery systems for efficient and selective gene therapies

Above all, the IDDSs with ionizable nanomaterials carrying corresponding charges at different pH values have achieved special effects during drug delivery, indicating their superior advantages in gene therapy [34]. Despite the progress made in gene therapy, there remains significant opportunity for improvement in the design of new IDDSs. To meet the needs of future development, we review the classifications and characteristics of IDDSs as well as the delivery mechanism and application in disease treatment as shown in Fig. 1. IDDSs can be divided into three types according to their different compositions showing different characteristics in biocompatibility, biodegradability, toxicity and manufacture properties, and their advantages/disadvantages are compared for improvement in the future. The factors affecting the transformation from formulation to clinic are analysed and rational design considerations for IDDSs are summarized to improve the efficiency of uptake and endosomal escape, achieve organ-selective delivery, and even reduce the application of auxiliary lipids to simplify the process complexity. The wide applications of the IDDSs are reviewed in some important fields, especially in two extensive research fields of virus infection and cancer currently. To explore more products of IDDSs for the medical market of gene therapy, it is anticipated that great efforts may be made on the high-throughput screening of ionizable nanomaterials by molecular dynamics simulation or artificial intelligence strategies.

## Ionizable drug delivery systems

**Fig. 2:**
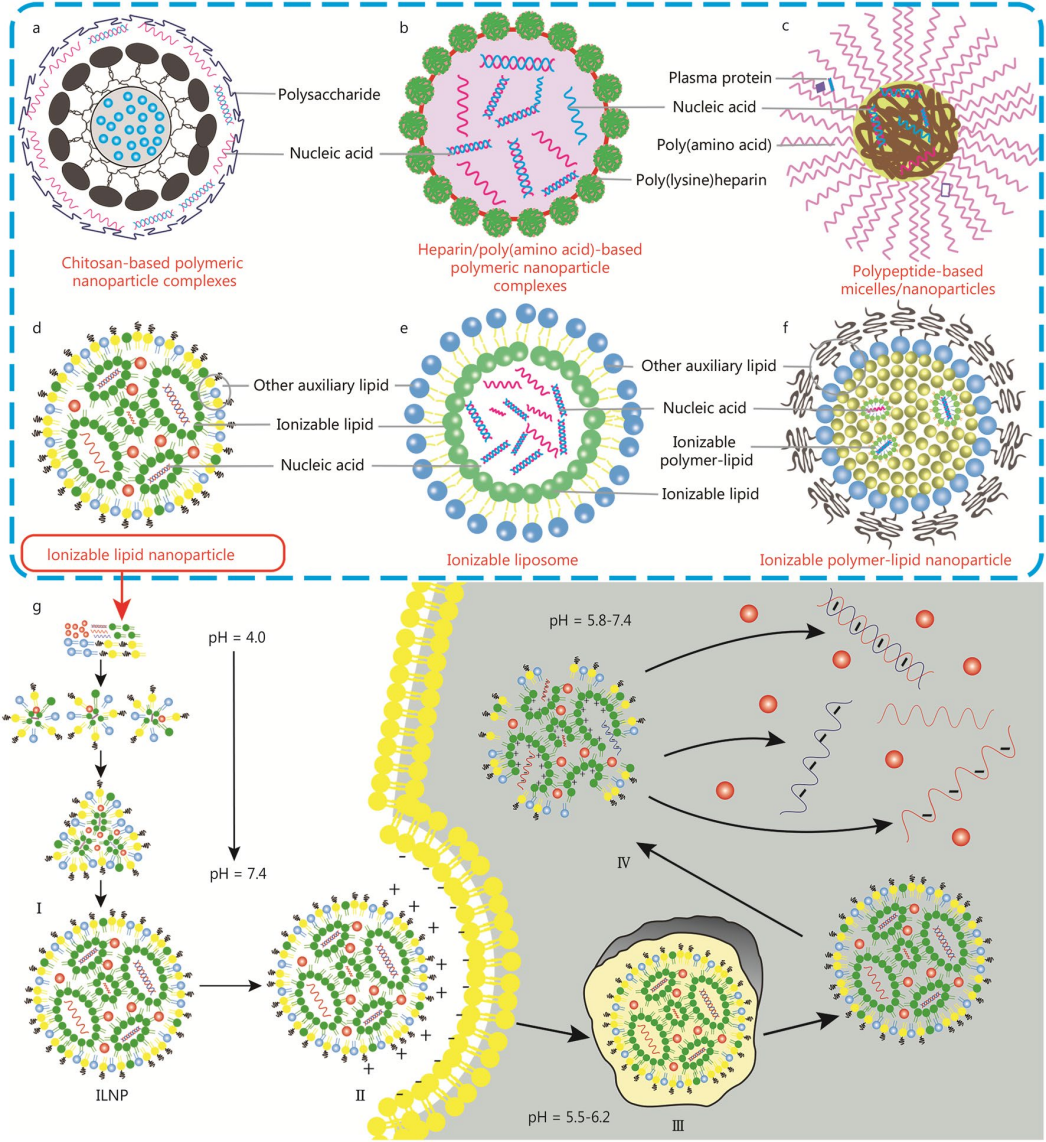
Classification of ionizable drug delivery systems (IDDSs). **a** Chitosan-based polymeric nanoparticle complexes. **b** Heparin/poly(amino acid)-based polymeric nanoparticle complexes. **c** Polypeptide-based micelles/nanoparticles. **d** Ionizable lipid nanoparticle (ILNP). **e** Ionizable liposome and **f** Ionizable polymer-lipid nanoparticle (IPLNP). **g** A hypothetical mechanism explaining the fate of IDDSs endosomes. I Formation of the lipoplex. II Binding to the cell membrane. III Uptake and endocytosis. IV Endosomal escape and release

IDDSs are generally divided into ionizable polymeric nanosystems, ionizable lipid-mediated drug delivery systems and ionizable polymer-lipid nanosystems. The types, compositions, advantages and disadvantages of the IDDSs are systematically summarized in Table 1. In particular, we introduce the compositions of six typical nanocarriers in IDDSs and demonstrate the delivery mechanism using ionizable lipid nanoparticles as an example as shown in Fig. 2.

**Table 1:** A representative summary of the applications of IDDSs in gene delivery

IDDSs	Vehicle	Composition	Payload	Advantage	Disadvantage	References
Ionizable polymeric nanosystems	Chitosan-based polymeric nanoparticle complexes	Chitosan/DNA	pDNA	Low cytotoxicity, biodegradability, low immunogenicity, low cost and high positive charge density	Transfection efficiency depends on a range of formulation parameters	[35]
		Chitosan/PLGA	OMR			[36]
		Chitosan/pGL3	pGL3			[37]
		Chitosan/DNA	pDNA			[38]
	Heparin/poly(amino acid)-based polymeric nanoparticle complexes	PDMAEMA/heparin	pDNA	They protect cationic polymers from interference by serum	Heparin itself cannot be used as a gene carrier	[39]
		Poly-l-lysine/heparin	siRNA and pDNA			[40]
	Polypeptide-based micelles/nanoparticles	Lipid/dendrimers	siRNA	Good biocompatibility and biodegradability. Amino acid residues can be infinitely combined and modified. Peptides have naturally encoded functions	The properties of peptide-based nanocarriers depend not only on peptides but also on lipids, polymers or inorganic components	[41]
		(HR)3gT peptide	ssDNA and dsDNA			[42]
		CR8GPLGVH5-Pal peptide	pDNA			[43]
		Bis(h9)-K-K4 and bis(h5)-K-K4 peptide	pDNA			[44]
		cRGD-hK peptide	pDNA			[45]
		RALA peptide	siRNA			[46]
		KHV-LHRH peptide	pDNA			[47]
Ionizable lipid-mediated drug delivery systems	Ionizable lipid nanoparticles	DODMA/eggPC/Ceramide-PEG	siRNA	Good biocompatibility, good biodegradability, low toxicity, low immunogenicity and structural flexibility. They are easy to manufacture on a large scale	They show some degree of instability in the blood	[33]
		Lipids with aminoglycoside tobramycin	mRNA, DNA and siRNA			[30]
		KALA peptide and ssPalmE-LNP	mRNA			[32]
		Ionizable lipid, Chol, DSPC and PEG-lipid	siRNA and mRNA			[48, 49]
	Ionizable liposomes	KC2, DSPC, Chol, and PEG-lipid	siRNA	They can increase the stability of the encapsulated drug. They enable transmembrane transport	They are prone to agglomeration, are unstable in storage, and have low drug loading	[50]
		Ionizable lipidoid, CH, DOPE, and PEG-lipid	mRNA			[51]
		DODAP, DOPE, DSPC, PEG-PE and Chol	siRNA			[52]
		MDH, DSPE-PEG and Chol	siRNA			[53]
Ionizable polymer-lipid nanosystems	PEI-lipid NPs	C15 epoxide-terminated lipids and PEI	siRNA	They enable non-immunogenic gene transfer and are easy to manufacture and store. They acquire various modifiable functions	Their endosomal release and targeting capabilities require further optimization	[54]
	PBAE-lipid NPs	PBAE and mPEG-PE	mRNA			[55]
	APE-lipid NPs	APE, Chol, and mPEG-PE	mRNA			[56]
	PSS-lipid NPs	PSS, amine-containing lipidoid	siRNA and mRNA			[57]

*PDMAEMA* poly(dimethylaminoethyl methacrylate), *DODMA* 1,2-dioleyloxy-N,N-dimethyl-3-aminopropane, *egg PC* egg phosphatidylcholine, *DSPC* 1,2-distearoyl-sn-glycero-3-phosphocholine, *Chol* Cholesterol, *DOPE* 1,2-dioleoyl-sn-glycero-3-phos-phoethanolamine, *DODAP* 1,2-dioleoyl-3-dimethylammonium-propane, *mPEG-PE* 1,2-distearoyl-sn-glycero-3-phosphoethanolamine-N-[methoxy(PEG)], *MDH* malate dehydrogenases, *PEI* polyethyleneimine, *PBAE* poly(β-amino ester), *APE* amino polyester, *PSS* polystyrenesulfonate, *NPs* nanoparticles, *siRNA* small interfering RNA, *mRNA* messenger RNA

### Ionizable polymeric nanosystems

Ionizable polymeric nanosystems are mainly based on polysaccharide complexes, polysaccharide/poly(amino acid)/heparin complexes, and amphiphilic polypeptides/poly(amino acid) complexes (Fig. 2). In this section, we systematically introduce and summarize each ionizable polymer nanoparticle.

#### Chitosan-based polymeric nanoparticle complexes

Polysaccharides derived from animals, plants and microorganisms have unique biological functions. Therefore, ionizable polymeric nanosystems based on polysaccharide complexes play an important role in gene therapy. Polysaccharides can be divided into homopolysaccharides (e.g., dextran and mannan) consisting of one repeating monomer and heteropolysaccharides (e.g., chitosan and heparin) with two or more repeating monomers [58–62]. We mainly discussed chitosan-based drug delivery systems in this section.

When the pH value is lower than the pK_a_ of chitosan, the primary amines in its backbone are positively charged, which makes chitosan have the potential to deliver nucleic acids. The presence of these protonated amines results in the ability of chitosan to bind to DNA/RNA in aqueous environments through electrostatic interactions and form nanoscale complexes [63]. In addition, chitosan can also bind to nucleic acids through hydrogen bonding and hydrophobic interactions due to its slightly charged properties in neutral or alkaline environments [64]. It has been reported that chitosan can concentrate nucleic acids to a size compatible with cellular uptake under a suitable nitrogen-to-phosphorus charge ratio [the ratio between chitosan nitrogen (N) and nucleic acid phosphate (P), N/P ratio], while the closed environment prevents the degradation of nucleic acid drugs by nucleases [35]. However, although the strong electrostatic charge is beneficial for nucleic acid loading, it may lead to difficulties in nucleic acid release at the target site. Therefore, formulation-related parameters need to be explored to achieve a balance between loading and releasing.

A number of cellular barriers have been reported for chitosan-mediated pDNA delivery, including enzymatic digestion, low cellular uptake efficiency, encapsulation in endolysosomes and failure of dissociation [65]. Previous articles have indicated that the affinity of chitosan to nucleic acids, the stability of chitosan/nucleic acid complexes, and the transfection efficiency depend on the molecular weight of chitosan, stoichiometry of the chitosan/nucleic acid complexes (e.g., nitrogen-phosphorus ratio and charge ratio), nucleic acid concentration, pH value of transfection environment and so on [36–38, 65–70]. The size of the complex decreased with decreasing molecular weight of chitosan [70]. It is reported that as the molecular weight of chitosan decreased from 213 to 48 kD, its average particle size reduced from 181 to 155 nm [35]. However, when the molecular weight of chitosan was further reduced to 17 kD and 10 kD, the average size of the nanoparticles reached 269 nm and 289 nm, respectively. These results suggested that an appropriate molecular weight of chitosan should be sought to obtain the suitable particle size since the transfection efficiency of nanoparticles is largely determined by the particle size [36].

The charge ratio of nanoparticles depends on the N/P ratio, which affects the interaction of chitosan with nucleic acids and the transfection efficiency of nucleic acid drugs [35]. As usual, there is a suitable range of N/P ratio for complexes used to deliver nucleic acids. For example, the chitosan/pGL3 complex obtained the strongest luciferase activity in A549 cells when the N/P ratio was 5:1 [37]. Romøren et al. [38] demonstrated that transfection efficiency increased with nucleic acid concentration and remained constant after reaching a critical point. It was found that luciferase expression increased when DNA concentration was increased from 0.5 to 2.5 µg/well, while it saturated when the DNA concentration rose up to 5 µg/well in EPC cells. Since the pH of the environment largely affects the charge density of chitosan, it affects the transfection efficiency of chitosan/nucleic acid complexes. It has been reported that a balance of binding and dissociation between nucleic acids and chitosan can be achieved at pH slightly below 7.0. In a pH 6.9 environment, A549 cells transfected more effectively than in a pH 7.6 environment [37]. This could be attributed to the fact that the chitosan complex was positively charged and could bind to cells via electrostatic interactions in an environment of pH 6.9. However, the transfection efficiency decreased in a more acidic environment due to the strong interaction of nucleic acids with chitosan, which ultimately resulted in slower nucleic acid release [68]. Changes in these influencing factors lead to different sizes, charge ratios, and release efficiencies of chitosan/nucleic acid complexes, resulting in different transfection efficiencies. As a result, these factors must be considered when preparing gene delivery systems.

#### Heparin/poly(amino acid)-based polymeric nanoparticle complexes

Heparin has a high net negative charge, which allows it to protect the cationic polymer from interactions with other blood cells. Therefore, it has been commonly reported that heparin and cationic polymers can be combined to lessen the cytotoxicity of the latter. For example, Nie et al. [39] synthesized a series of heparin-based comblike copolymers grafted with cationic poly(dimethylaminoethyl methacrylate) (PDMAEMA) and tested their effectiveness for transfection and cytotoxicity. They adjusted the solubility of heparin from water to oil by introducing a tetrabutylammonium group. Heparin-based comblike copolymers with PDMAEMA grafts were then readily prepared by free-radical polymerization in methanol/dimethyl sulfoxide mixtures. Negatively charged heparin and PDMAEMA grafted then self-assembled to form polymeric nanoparticles through electrostatic interactions. The in vitro cytotoxicity assay results showed that the complex was less cytotoxic to HepG2 cell line compared to polyethyleneimine (PEI, 25 kD) and PDMAEMA (Hep-PD) alone, suggesting that the cytotoxicity of the cationic carrier may be inhibited by the negatively charged properties of heparin. The transfection efficiency of PDMAEMA/heparin complexes was tested on HepG2 cells. Compared with controls (PEI and PDMAEMA), the heparin-based vector was more efficient in transfection in HepG2 cells, which may be attributed to the lower cytotoxicity of the complex and the degradation of the heparin backbone in cells by heparinase.

While polycations offer the opportunity for polymeric nanoparticles to bind to DNA and penetrate cells, and they do not result in efficient release of genetic structures into the cytoplasm [71]. However, polyanions can facilitate the release of genetic material from polymer nanoparticles through competitive interactions with polycations [72]. In recent research, the delivery of siRNA and pDNA was achieved by creating polymeric nanoparticles of poly-L-lysine with heparin [73]. A photosensitive linker (4-bromomethyl-3-nitrobenzoic acid) was subsequently added for photosensitive release of genetic material [40]. The ratio of heparin was required to be greater than that of polylysine in the nanoparticles to facilitate the release of genetic material. The outcomes indicated that the nanosystems obtained photoactivation of siRNA and pDNA transfection.

#### Polypeptide-based micelles/nanoparticles

The properties of peptides (e.g., hydrophilicity, hydrophobicity and charge) allow them to deliver genes in biological systems. Peptide-based micelles are self-assembled from a hydrophobic core and a hydrophilic outer shell. In terms of gene delivery, peptide-based micelles have the benefits of effective gene loading and suitable size, which allows them to penetrate deeply into tumors and be taken up by cells [74]. In particular, the formation of cationic micelles in solution causes an increase in the density of positive charges, which results in efficient condensation of genetic material [74]. In a study, cationic peptides with lipid-conjugated side arms were shown to be able to self-assemble into micelles and simultaneously condense with siRNA [41]. Particularly, the results showed that the peptide-based micelles were not cytotoxic under any conditions and their transfection efficiency was superior to other common reagents (e.g., Lipofectamine 2000). Similarly, amphiphilic peptides were shown to self-assemble into multi-compartmental micelles and efficiently capture DNA with chains of approximately 100 nucleotides in length [42]. Cationic peptide-mediated micelles are capable of condensing nucleic acids and modulating the efficiency of the parameters affecting transfection (e.g., cellular uptake and endosomal escape) by modulating various parameters of the cationic peptide [74]. For example, a multifunctional dipeptide (CR8GPLGVH5-Pal) containing a matrix metalloproteinase 2-responsive sequence (GPLGV), a cell-penetrating moiety (R8), and a pH-responsive moiety (H5) is self-assembled into micelles and could be condensed with pDNA [43].

Nucleic acid/peptide nanoparticles can be formed by electrostatic interactions between cationic peptides and phosphate backbones with anions. Genetic material is able to be condensed using positively charged peptides with lysine, histidine, and arginine residues. For example, lysine-rich peptides could condense with genetic material based upon the genetic payload concentration [75]. Similarly, amphiphilic peptides containing oligolysine fragments were able to condense into nanoscale complexes with green fluorescent protein encoding pDNA due to the strong interaction of oligopeptides with pDNA in a certain ratio [44]. As shown in the results, the nanocomplexes transfected HeLa cells to a higher degree than the transfection reagent lipofectin. Globular peptides with lysine end groups and poly(l-leucine) conjugated with glutamic acid residues were also demonstrated to be suitable for gene delivery due to its self-assembling into nanoparticles [76].

The other peptides with rich positively charged amino acids such as histidine and arginine have also been explored. It has been shown that short peptides with multiple histidines increased the effectiveness of DNA transfection possibly due to its protonation at low pH facilitating endosomal escape [45]. Arginine-containing peptides in a similar manner have been shown to have the ability to condense with genes and facilitate cell penetration [77]. One example reported by Benett et al. showed that an amphiphilic cell-penetrating peptide (RALA) containing 7 arginines condensed with siRNA and pDNA to form complexes for gene delivery [46, 78].

Compared with the single use of individual basic amino acids, the combination use of arginine and histidine can enhance transfection efficiency due to a stronger cell penetration ability [79]. Amphiphilic peptides formed by the polymerization of various amino acids have also been gradually developed. In a cationic amphiphilic peptide (K12H6V8), lysine could be condensed with DNA, histidine was used for endolysosomal release, and valine was used as the hydrophobic moiety [47]. The luteinizing hormone-releasing hormone ligand-modified cationic peptide had higher transfection efficiency and gene expression in MCF-7 cells than the unmodified cationic peptide.

### Ionizable lipid-mediated drug delivery systems

Common ionizable lipid-mediated drug delivery systems are composed of ionizable nanomaterials, other auxiliary components, and nucleic acids (Fig. 2). In this section, we mainly introduce and summarize ionizable lipid nanoparticles (ILNPs) and ionizable liposomes.

#### Ionizable lipid nanoparticles

ILNPs are mainly composed of ionizable lipids with pH-responsive properties and auxiliary components (e.g., cholesterol, PEG, and phospholipids) that exert diverse roles. In this section, we provide a systematic introduction to the common components of ILNPs.

The critical composition of ILNPs is ionizable lipids with pH-responsive properties. Ionizable lipids that are stable in neutral pH can connect with nucleic acids to achieve more destabilized complexes in acid environment, which are highly desirable for endosomal escape [80]. For instance, Wang et al. [33] disclosed that 1,2-dioleyloxy-N,N-dimethyl-3-aminopropane (DODMA) were strongly depended on the tertiary amine to achieve pH-responsive property. In physiologically neutral environments, positively charged IDDSs could be more stably attached to nucleic acids and enhance interactions with cell membranes, which facilitated cell uptake by mediated endocytosis. Only in acidic environments do IDDSs dissociate and release the cargo upon contact with the acidic component of the endosome. In addition, Habrant et al. [30] investigated a 4,6-disubstituted 2-deoxystreptamine derivative composed of various ionizable lipids with aminoglycoside tobramycin. The structure of aminoglycosides with several amino groups (together with numerous hydroxyl functions) provided a versatile polycationic framework, so it can stably bind to nucleic acids. IDDSs based on the aminoglycoside tobramycin had shown promising results in mRNA delivery [30, 33].

The amphiphilic structure of ionizable lipids can be generally divided into three parts: ionizable headgroup, hydrophobic domain, and linker area that joins the two parts to form ILNPs [81]. Structural composition can cast a significant impact on drug delivery performance. For example, ILNPs based on the vitamin E-scaffold (ssPalmE) have been developed with the 3 above-mentioned structural parts of an SS-cleavable and pH-activated lipid-like material (ssPalm) as a hydrophilic ionisable head, two SS-cleavable amphiphilic motifs as the hydorphobic scaffolds, and the ester groups as the linker part [32]. Owing to dual-sensing features, ssPalm successfully delivered nucleic acids into the cytoplasm of the hydrophilic head. The hydrophilic head was composed of tertiary amines and a disulfide bond, enabling endosomal escape and cytoplasmic release. Moreover, Ramishetti et al. [48] designed a novel aminolipid molecule by mixing ethanolamine, hydrazine, or hydroxylamine with appropriate methods. They chose active linoleic acid chains or branched lipid chains with unstable ester as hydrophobic chains, and the tertiary amines are ionizable heads. The precise and rational design of the compositions and degree of unsaturation of lipids are necessary to further enhance the delivery efficiency [32, 48].

Cholesterol, as one of the auxiliary components of ILNPs, enhances the stability of ILNPs and promotes membrane fusion [82]. The relatively low solubility of cholesterol meant an enrichment of cholesterol in the bulk phase, which might allow the formulation of cholesterol to crystalline on the surface of ILNP [49]. These visible crystals on ILNPs have been studied, and the results show how crucial a function they play in facilitating endosomal escape. Furthermore, the multivalent and membrane rigidity of carrier-target membrane surface interaction might be led by the differences of cholesterol distributed laterally on the membrane surface and differences in membrane mobility [83]. Therefore, cholesterol was a decisive cofactor that modulated the interaction of lateral phospholipids with proteins, and a function of curvature that provided vesicle assembly by promoting membrane bending and invagination [84].

For improving the stability and integrity of ILNPs, the hydrophilic polymer-lipids [e.g., polyethylene glycol-lipids (PEG-lipids)] were used to reduce the aggregation and nonspecific endocytosis of ILNPs by immune cells [82, 85, 86]. A PEG-lipid can control the particle size of ILNPs [87] and improve the lipid membrane stability during incorporation, resulting in effective prevention of aggregation, degradation, and opsonization [86] for better preservation [88]. Kulkarni et al. [50] found that the outer monolayer would appear through further fusion if the PEG-conjugated lipids were not present in the nanoparticles. This study involved a large number of amphiphilic lipids and controllable additional fusions. Though amphipathic lipid is not adequately contained to cover surface coating, it exhibits a stable metastable state on the PEG-lipid outer surface. PEG not only enhances the stability of ILNP, but also affects the half-life of the drug. The ILNPs accumulated only in diseased cells and required a long circulating half-life to effectively cure the disease [89]. This can be achieved by reducing opsonization with PEG.

In particular, the bilayer structure of ILNPs is favourable for their endosomal escape [90], which also contributes to the formation of multilamellar vesicles and the enhancement of the stability of ILNPs [91]. A small amount of phosphatidylcholine is used to support stable ILNPs during the formation and recycling of ILNPs [87]. Unsaturation of the lipid tail promoted the formation of a hexagonal bilayer, leading to more efficient RNA release. As Liu et al. [24] pointed out, phospholipids facilitated the complexation of mRNAs with ILNPs, leading to improved delivery efficiency [24]. Moreover, phospholipids showed the resemblance to biological membranes, which might enable membrane fusion rapidly.

#### Ionizable liposomes

Ionizable liposomes are remarkable IDDSs, which have no charge in the blood circulation, but are protonated in lower pH environments (e.g., endosomes and lysosomes). These molecules work with apolipoprotein E (ApoE) and are transferred to the liver. The major ionizable liposomes are asymmetric liposome particles (ALPs), O’^1^, O^1^-[3-(dimethylamino) propane-1,2-diyl] 16-bis[2-(2methyl-5-nitro-1H-imidazol-1-yl)ethyl] di(hexadecanedioate) liposomes (MLP), large unilamellar vesicles (LUVs) and others.

ALPs contain the siRNA inside and the PEGylated lipid outside, which ensures efficient siRNA encapsulation [52]. However, ALPs are not easy to internalize into cells. This problem can be solved after being modified by a target-specific antibody (anti-EGFR) and generating siRNA-dependent gene-silencing effects.

MLP utilizes electrostatic interaction to pack siRNA. In physiological environments (pH 7.4), the positive charge density of tertiary amines on the surface of MLPs remains to be in a low range, allowing them to provide longer half-lives in circulation. In the tumor tissue, the tertiary amine head groups are protonated at acidic pH (6.8–6.5), increasing the permeability of the MLPs with more positively charged density into cells [53].

pH-Sensitive fusion of LUVs was adjusted by anionic lipid component cholesteryl hemisuccinate. At the low pH, DODAC/cholesteryl hemisuccinate LUVs could constitute stable bilayers, whose ionizable lipid was charged. DC-cholesterol with a pK value of 8.0 was an ionizable cationic lipid, while DOPA with pK of 3.0 was chosen as ionizable anionic lipid and fused as the pH was enhanced. This behavior was put down to augment protonation between DC-cholesterol and DOPA, resulting in a reduction in surface charge [92].

Ionizable liposomes with transmembrane pH gradients exhibited long-circulation properties for the therapy of calcium channel blocker poisoning. The characteristic of acid internal compartments was effective for ion-trapping weakly alkaline drugs. The rationale for detoxification of liposomes through transmembrane pH gradients was to fully exploit their properties to achieve free drug capture [25]. This study mainly examined whether the ionizable materials synergistically accomplished gene silencing. It was found that combining two ineffective siRNA drugs with IDDSs promoted protein silencing using liposomes as a model delivery system [93].

### Ionizable polymer-lipid nanosystems

The IPLNPs composed of a polymer core and a lipid shell possess complementary properties of polymer nanoparticles and lipid nanoparticles in terms of physical stability and biocompatibility. It is worth noting that IPLNPs have recently been shown to present superior cell delivery efficiency in vivo compared to lipid nanoparticles and liposomes [84].

The IPLNPs can transfer mRNA or siRNA effectively. For example, Dahlman et al. [54] identified that PEI could be used to deliver nucleic acid-based cargoes and developed a formulation called 7C1, which could deliver siRNA to lung endothelial cells at low doses without substantially reducing gene expression in pulmonary immune cells, hepatocytes, or peritoneal immune cells. In addition, poly(β-amino ester) (PBAE) nanoparticles formed by phospholipid bilayers and PBAE cores loaded mRNA onto their surface through electrostatic interactions and delivered mRNA vaccines via nasal mucosal administration [55]. PBAE was selected as the polymer core due to its inherent pH-responsiveness that could promote the destruction of internal lysosomes. The role of lipids is to impart biocompatibility to the PBAE core and to mediate mRNA adsorption. The results of in vitro experiments showed that the complexes could be easily absorbed by dendritic cells and transferred to the cytoplasm of dendritic cells to minimize toxicity. Moreover, the production of ring-opening polymerization (ROP) involving amine polyesters for nucleic acid delivery has also been disclosed [56]. However, ROP was mutually incompatible with primary and secondary amines. The researchers chose ionizable amino polyester as ionizable lipids to avoid the problems of conversion rate, scalability, and polymer chain degradation caused by post-polymerization modification of functionalized polyesters or other reasons [56]. The ROP of lactones with tertiary amino alcohols in turn led to amino polyester, which is important for delivering mRNA to tissues and cells.

Recently, polypeptide poly(glycolic acid) (PGA)-based IDDSs have also been developed [26, 94]. PGA was a bioinspired hydrophilic synthetic peptide polymer capable of acting as a scaffold for covalent attachment of peptide antigen (Ag) [26]. IDDSs can be formed by electrostatic interaction between PGA and ionizable cationic lipids. They can further be absorbed by antigen-presenting cells via lymphatic transport, thereby inducing maturation of CD8^+^ T cells through the cancer immune cycle. The IPLNPs composed of lipids and PGA were also designed to load therapeutic siRNA against tumor necrosis factor (TNF) [94]. The co-transmission of mRNA and siRNA employed the lipids with polystyrenesulfonate (PSS) containing additional negative charges [57]. More stable nanoparticles were formed by electrostatic attraction and then showed high delivery efficiency of nucleic acid both in vivo and in vitro. In particular, a small-scale study carried out by Zhang et al. [95] indicated that a novel amino lipid, including an aliphatic tail and a cholesteryl ether tail, could be used as an ionizable amine headgroup for the IDDS delivering siRNA. Attaching the lipid tail to the steroid structure reduces the lipid exchange, decreases membrane permeability, and weakens protein adsorption to the lipid membrane.

The following factors may be worth taking into account when choosing lipid components for nanoparticles: (1) Biocompatibility. Natural lipids are preferred and the most widely used lipids are phospholipids. (2) Liquidity. Cholesterol can regulate the fluidity of lipid bilayers, with solid bilayer fluidity rising and liquid bilayer fluidity decreasing. (3) Phase state. Phase states are related to the stability and encapsulation efficiency of ILNPs, and govern interactions with biofilms and content release. (4) Charge (zeta potential). The charge of ILNPs affects their stability, release rate, circulating half-life, and biofilm interactions. (5) Toxicity. Toxicity is particularly relevant for formulations containing synthetic cationic lipids. (6) Particle size. Size is a key parameter determining the drug encapsulation of ILNPs. (7) Circulation time. Coating ILNPs with inert polymers can extensively extend their residence time in blood circulation. (8) Content release. ILNPs must be stable enough to convey the contents to the target while being able to decompose in time to release the contents in the target area. (9) Packaging efficiency and stability. Replacing conventional liposomes with nanostructured lipid carriers can improve the encapsulation efficiency of nanoparticles.

The aggregation states of ionizable lipids in an aqueous environment will obtain various structures with different concentrations, mainly including micellar phase, lamellar phase, and inverted hexagonal phase etc.. The different phase states can be predicted by *P* = V/alc (V is the volume of amphiphilic lipids, a is the head group area, and lc is the critical tail length). When *P* > 1, inverted hexagonal lipids can be formed and promote the fusion of lipid membrane, which destabilize the endosomal membrane and accelerate endosomal escape [96].

It is not only the phase state but also the particle size of IDDSs affect the drug delivery efficiency. The size of IDDSs is usually 20–200 nm, which is relatively robust and can withstand the flow of blood. This size can also pass through the intercellular substance. What’s more, different applications may require various particle sizes. For example, 45 nm siRNA-IDDSs are most effective for subcutaneous administration; while 80 nm siRNA-IDDSs are suitable for intravenous injection [97]. As reported, there is a strong correlation between the particle size and encapsulation efficiency of IDDSs, and the N/P ratio (tertiary amine in ionizable lipids/phosphorus in nucleic acids) influences the nucleic acid loading capacity of IDDSs [85, 98]. N/P ratio is one of the factors that affect the encapsulation efficiency of nucleic acid drugs. Fan et al. [85] mainly studied the effect of N/P ratio on encapsulation efficiency of antisense oligonucleotides. When N/P ratio is greater than 1, the encapsulation ratio is greater than 80%. However, when the N/P ratio is reduced to 0.5, the encapsulation ratio is significantly reduced to 50%. N/P ratio also affected the electrification of nanoparticle, and then affected the encapsulation efficiency of drugs [98]. The particle size of IDDSs is around 120 nm to enter the cell through clathrin-mediated uptake and endocytosis mechanism and play a role [98, 99].

Although the chemical modification of ionizable materials can improve efficacy and reduce off-target effects and side effects, the safe and efficient vectors are undoubtedly a key factor in promoting the approval and marketing of genetic drugs. It is worth mentioning that the crucial factors that lead to the FDA-approved mRNA vaccine’s successful creation for the treatment of coronavirus also involve efficient and safe delivery vehicles. With the continuous in-depth researches of biotechnology and delivery vehicles, clinical illness treatment in the future will get greater promise for nucleic acid therapy.

## Classification of nucleic acid delivered

**Fig. 3:**
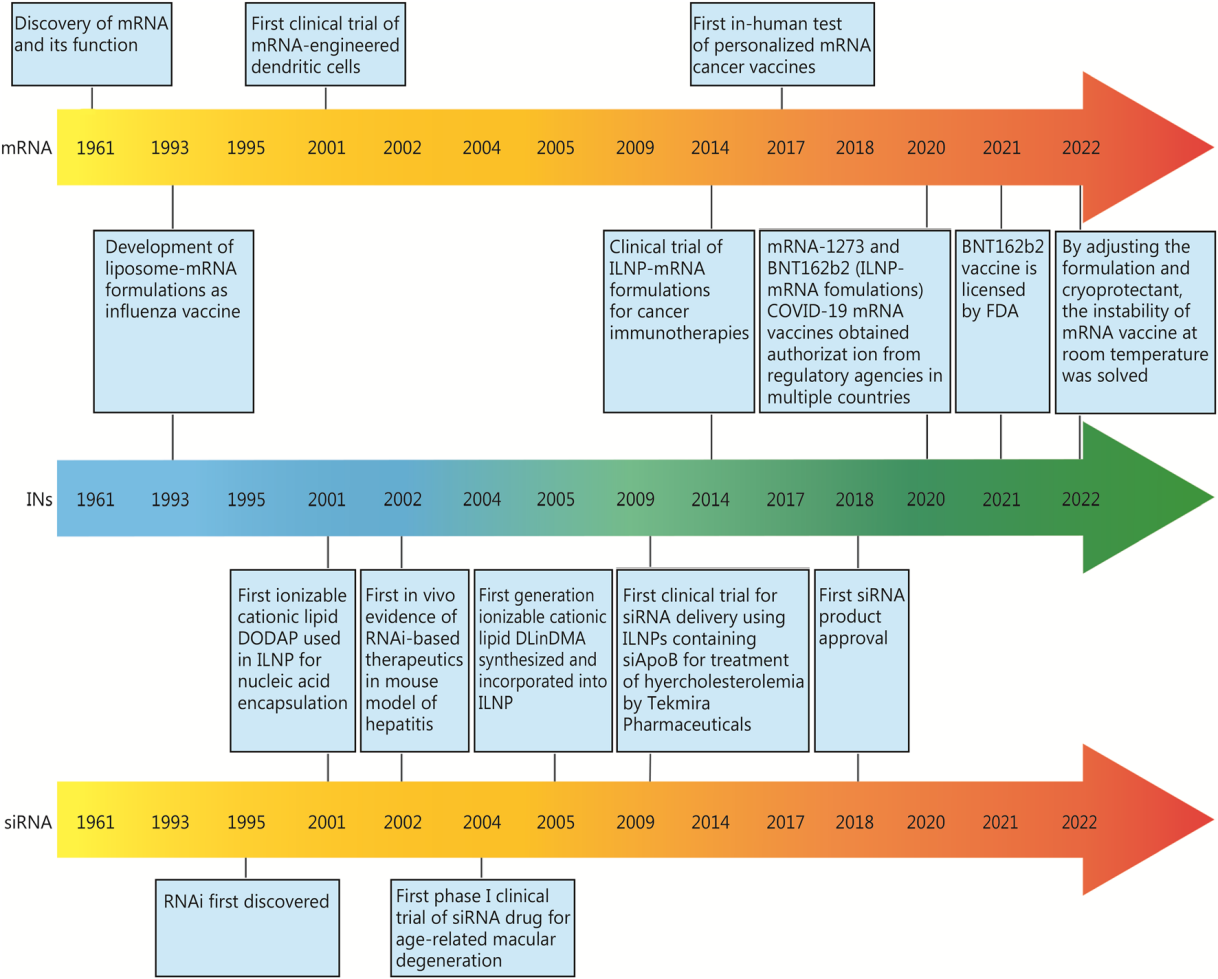
Discovery and progress schedule of nucleic acid technology and applications. FDA Food and Drug Administration, ILNP ionizable lipid nanoparticle, RNAi RNA interference, mRNA messenger RNA, siRNA small interfering RNA, COVID-19 coronavirus disease 2019, DODAP 1,2-dioleoyl-3-dimethylammonium-propane, DLinDMA 1,2-dilinoleyloxy-N,N-dimethyl-3-aminopropane

The drugs delivered by IDDSs are nucleic acids, mainly including siRNA, mRNA, sgRNA, saRNA, and pDNA, among which siRNA and mRNA are the earliest, most studied and most widely applied (Fig. 3).

### siRNA

siRNA with a short double-stranded structure containing about 22 nucleic acids expresses and silences genes in a sequence-specific manner [100]. Even though it has been proposed in clinical trials, siRNA faces difficulties in entering cells because of its high molecular weight and negative charge [33]. Fortunately, IDDSs-siRNAs that silence disease-causing genes after intravenous injection have recently passed a clinically validated system. In order to analyse the mechanism of IDDSs-siRNA, researchers have carried out a large number of experiments. For instance, the formation mechanism and certain structural features of ILNP-siRNA have been revealed by Kulkarni et al. [50]. At pH 4, only small vesicles were formed. As the pH increased to 7.4, the particles fused to the particles. After the fusion of lipid nanoparticles reached equilibrium, they further bound to siRNA and formed IDDSs. A cationic helical polypeptide-based hybrid nanoparticle has also been developed for the delivery of siRNA of TNF-α [101]. Helical structures can form pores in endosomal membranes to promote endosomal escape of TNF-α siRNA in immune cells. Nanoparticles containing TNF-α siRNA exhibit potent knockdown of TNF-α and exert potent anti-inflammatory effects after systemic administration.

At present, ALN-VSP and TKM-PLK1 are both siRNA-IDDSs-based therapeutic approaches that have been quite effective for solid tumors in clinical trials. With the dosage increasing from 0.1 to 1.5 mg/kg, ALN-VSP and TKM-PLK1 showed well tolerance compared with chemotherapy or other therapies. In patients’ tumors, the accumulation of siRNA along with target gene-specific silencing could be detected. In clinical phase I study, ALN-VSP and TKM-PLK1 had good stability and safety [102]. According to the analysis of tumor biopsy, ALN-VSP had good tolerance and clinical activity. Through RNAi therapy, ALN-VSP directly inhibits cells from producing harmful proteins. At higher doses, some patients experienced mild to moderate chills [103]. While TKM-PLK1 inhibits cells from proliferating, which causes tumor cells to perish. However, the unsatisfactory antitumor effect of TKM-PLK1 is worrying [104].

IDDSs can solve the challenge of how to ensure highly efficient delivery to keep high-efficiency siRNA for systemic administration, which leads to great interest in siRNA as a new therapeutic tool [105]. Patisiran, the first siRNA drug developed by Alnylam for the therapy of hATTR amyloidosis, was authorized by the FDA in 2018. Unlike Inotersen’s mechanism of action, Patisiran is delivered via ILNP. Inotersen is an antisense oligonucleotide drug for the therapy of hATTR amyloidosis by delivery into the nucleus. The continuous accumulation of transthyretin (TTR) protein in hATTR amyloidosis patients can lead to movement disorders. Inotersen mainly targets blocking the production of TTR protein to treat the disease, but the drug is a single-stranded nucleotide sequence that binds to other mRNAs and interferes with its activity [106]. It also induces gene silencing in combination with expressing wild-type and mutant TTR mRNA and reduces TTR protein production, thereby improving amyloidosis accumulation.

siRNA with hydrophilicity has weak binding ability to plasma proteins, which makes it clear faster in plasma. Therefore, there is a greater need for suitable delivery systems and chemical modifications to enhance their pharmacokinetic and biodistribution qualities in vivo. The four siRNA drugs currently on the market have adopted the delivery system of ILNP or GalNAc (N-acetylated galactosamine). After siRNA is taken up by cells, they should escape from endosomes to reach the cytoplasm, or they would be degraded by nucleases in lysosome. In systemic administration, a targeted drug delivery system can be created to target the focal organ to play a role. Patisiran needs to be filtered by a 0.45 µm PES filter according to the prescribing information prior to infusion. Because if Patisiran is filtered directly from the vial, the lipid nanoparticles within it will experience shearing phenomenon as a result of the increase in pressure, preventing the drug’s active ingredient from entering the cells.

### mRNA

mRNA was first discovered in 1961 and was considered for drug research by Malone [107] in 1989. In 1990, mRNA was transfected and expressed in mouse skeletal muscle cells under the entrapment of ionizable lipids, proving the feasibility of mRNA vaccine development [108, 109]. After that, mRNA delivery technology became a difficult problem in the development of mRNA vaccines, which constituted a technical barrier for mRNA vaccine manufacturers. Recently, mRNA vaccines have gradually entered the clinical trial stage. The emergency use of the COVID-19 mRNA vaccine was authorized by FDA in the United States in 2020, and the next year, the COVID-19 mRNA vaccine was officially approved by FDA [110, 111].

At pH 5.0, IDDSs with branched tail lipids have strong ionization intensity and can deliver rapidly mRNA in mice. For ionizable lipid-mediated drug delivery systems, siRNA delivery systems depend on the pK_a_ of lipid nanoparticles, whereas mRNA delivery systems depend on charged lipid nanoparticles [51]. mRNA transport to target cells is dependent on IDDSs due to hydrophilicity, large size and strong anionic charge inhibiting the passive diffusion function of cell membranes. The efficacy of the mRNA delivery system depends on the components (e.g., unsaturation and tail length of lipids) within IDDSs [112]. Despite being less developed, ionizable polymeric nanoparticles have advantages, such as high stability and tunable surface properties. For example, the chitosan-based drug delivery system’s transfection effectiveness is reliant on the balance between various parameters. The endosomal escape ability of chitosan nanoparticles needs to be significantly enhanced to compete with currently available clinically relevant ILNPs. Poly(2-propylacrylic acid) (PPAA) was added to chitosan/mRNA nanoparticles to enhance transfection efficiency [113]. Due to the inherent membrane-solubilizing ability of PPAA, the nanoparticles with the optimal formulation achieved an expression level of 86% of that of commercial lipids at pH 6.5.

### sgRNA

sgRNA is a single-stranded RNA with about 20 nucleotides in length. It is the nucleic acid portion of the CRISPR genome-editing tool. CRISPR/Cas9 is one of the most popular gene-editing tools. The CRISPR/Cas9 gene-editing system includes sgRNA and Cas9 nuclease. sgRNA guides Cas9 nuclease to perform double-stranded cleavage at specific gene loci, thereby correcting disease-causing genes or introducing beneficial genes to achieve the purpose of treating diseases. In particular, CRISPR/Cas9 gene can permanently interfere with tumor survival genes to overcome limitations of traditional cancer therapy. The catalytic domain in the Cas9 nuclease cleaves chromosomal DNA into blunt-ended DNA with double-strand breaks (DSBs). DSBs induce insertion or deletion of genes primarily through error-prone non-homologous end joining [114]. Homology-directed repair is also an alternative possible mechanism to repair DSBs. Compared with physical and viral delivery, non-viral delivery of the CRISPR/Cas system has unique advantages in terms of safety, loading capacity, and preparation. Therefore, many researchers focus on developing non-viral vectors with high delivery efficiency, which is important for the application and translation of promising technologies. Recently, IDDSs for delivering sgRNAs have gradually been developed to treat cancer. For instance, an ILNP based on iLY1809 lipid containing psgPLK1 expressing sgRNA targeting Polo-like kinase 1 was developed [115]. It achieved more potent tumor growth inhibition compared to siRNA. Additionally, cationic α-helical polypeptide-based PEGylated nanoparticles were developed for the delivery of sgRNA and Cas9 expression plasmids [116]. Due to the presence of cell-penetrating α-helical polypeptides, the ionizable polymeric nanosystems achieved efficient endosomal escape, reaching 47.3% of intracellular gene editing and potent tumor growth inhibition. Compared with existing polycationic transfection reagents, the CRISPR/Cas9 delivery system has great potential for knock-in and gene activation and can be further extended to gene editing in vitro and in vivo.

### saRNA

saRNA is double-stranded RNA and its structure is similar to siRNA, but the effect is different. The mechanism of action of saRNA to activate gene expression is related to Ago (argonaute) proteins and histones in nucleosome structure. It is capable of inducing protein expression for 60 d, which has served as a vaccine resisting multiple diseases (e.g., infectious diseases and cancer). saRNA induces an immune response in humans at low doses and is considered as a promising current biotherapeutic tool. It is a negatively charged macromolecule (approximately 9500 nT) [117] and requires IDDS as a carrier to encapsulate RNA in particles for cellular uptake and degradation protection. To date, the delivery vehicles for saRNA that have been developed are ILNPs, complexes and cationic nanoemulsions. With the approval of the COVID-19 mRNA vaccine, ILNP is currently the most widely used clinically. However, IDDSs for the delivery of saRNA vaccines are still under further development and research. Polymeric (pABOL) and ILNP delivery systems for saRNA vaccines were also investigated [118]. The results showed that the intramuscular protein expression of pABOL preparation was 100-fold higher than that of ILNP. However, ILNP had higher antibody and cellular responses to influenza and SARS-CoV-2 antigens compared to pABOL. This study indicated immune sensing of ionizable materials and the effect of saRNA on formulation efficacy. They also found that cationic lipid-based ILNPs could prevent saRNA from being degraded by RNAse even when it was on the surface [117]. Besides, saRNA-loaded cationic ILNPs or ionizable lipid particles exhibited the same delivery efficiency in vivo.

### pDNA

pDNA is a small circular double-stranded DNA molecule showing important research potential in gene therapy [119]. It often carries genetic sequences that may be beneficial to the survival of the organism. Besides, it is also often regarded as a replicon and can replicate autonomously in different host organisms. In genetic engineering, artificially constructed plasmids are often used as carriers for special genes. The modified pDNA can be used to compensate and correct the missing protein in cells at genome level. Compared with the gene drugs (e.g., siRNA and mRNA) that are released into the cytoplasm to exert their effects, pDNA must pass through the nuclear pore and enter the nucleus to achieve expression. When the necessary pDNA is transported to the nucleus, the barrier effect of the double-layered nuclear membrane structure prevents the pDNA from entering the nucleus. For nerve cells, it is difficult for an undivided neuronal cell to express any transgene, even if it is just a single particle. Even in the adult brain, mature neurons can undergo cell division unlike neural stem cells and neural precursor cells. Since the nuclear transfer of pDNA as well as gene expression were complicated, neural stem cells or neural precursor cells exhibited uptake of gene target delivery when the nuclear membrane decayed during the mitotic phase. For example, IDDSs consisting of positively charged ionizable amino lipids (YSK-MEND) were used to deliver pDNA [88]. Based on the analysis of fluorescently labeled ApoE/YSK-MEND and expression of fluorescent protein (mCherry), this delivery system with pDNA could promote transgene expression, suggesting that the formulation was a potential biological tool for neurodegenerative diseases. Furthermore, Kimura et al. [88] developed pDNA nanocarriers for improved transfection in splenocytes driven by synergy between an octaarginine (R8) peptide and an ionizable amino lipid (YSK05). After optimization, the R8/YSK system exhibited high gene expression in the spleen with significant capability to target splenic B cells. Significant tumor growth inhibition was exhibited in mice immunized with the R8/YSK system loading antigen-encoding pDNA. In recent work, a small ILNP library for pDNA delivery in cardiomyocytes was developed [120]. The optimized ILNP induced a two-fold increase in the expression of ionizable drug delivery systems in cardiac tissue compared to the control group.

There are several advantages about mRNA compared to pDNA. mRNA might achieve the translation in the cytoplasm and be completely metabolized without the risk of genome integration in the whole process, while DNA-based therapeutics showed drawbacks in this connection. Due to unformulated nucleic acids never achieving enough levels of gene expression, an essential question for the effective application for nucleic acid treatment was the further promotion of nucleic acid entry into host cells. The high-efficiency transfer of DNA had been demonstrated in viral vectors, but they still presented unacceptable immunological responses and the specific genes that were transported remained restricted [121]. Gomez-Aguado et al. [122] have developed different solid lipid nanoparticles (SLNs) for the delivery of mRNA and pDNA. They also studied the effect of SLN preparations on transfection in human retinal pigment epithelial cells (ARPE-19) and human embryonic kidney cells (HEK-293). In ARPE-19 cells, comparing the mRNA preparation to the pDNA preparation, a higher proportion of transfected cells were induced. However, the pDNA preparation induced cells to produce more protein in this cell line [123]. Cell line, SLN composition, and the type of nucleic acid supplied all had an impact on protein production.

Nucleic acid drugs can regulate the genes expressing related proteins based on the principle of base complementation, rather than binding to target proteins. It can also enter cells to function through suitable delivery systems. Therefore, nucleic acid drugs can avoid the limitation of undruggable targets faced by traditional small molecule drugs and antibody drugs. Even so, the development of nucleic acid drugs has gone through a long process, and its instability, immunogenicity, low cellular uptake efficiency, and difficulty in endosomal escape have limited the development of nucleic acid drugs. However, breakthroughs in key technologies have played an important role in improving the above-mentioned defects, including chemical modification and delivery systems.

## Delivery mechanisms of IDDSs

Recently, several excellent reviews proposed pH-sensitive drug delivery systems, but they tend to focus on pH-sensitive nanosystems mediated by acid-cleavable chemical bonds [124], cationic lipids and polymers for mRNA vaccine delivery [125], and pH-sensitive polymeric micelles for tumor-targeted delivery [126]. In this review, we provide a detailed introduction to IDDSs in terms of materials, nucleic acid delivery, and disease applications. In particular, we discuss the delivery mechanism in depth. IDDSs improve the delivery efficiency of nucleic acids through a unique delivery mechanism. As shown in Fig. 2, we take ILNPs as an example. First, before entering the cell, the cationic lipids achieve complexation with the negative charged nucleic acid, which improves the stability of nucleic acid. Second, when the complexation with nucleic acid/IDDSs reaches the cell membrane, the cationic lipids bind to the negatively charged cell membrane to promote the delivery of nucleic acid into the cells through destabilizing membrane [18]. Then, the nucleic acids/IDDSs enable endosomal escape and release.

Endosomal escape is thought to be necessary to avoid nucleic acid degradation and its efficient transfection. After being endocytosed into the cell, the complex fuses with the endosomal membrane and is encapsulated in the early endosome. And the nucleic acid drug is released by endosome escape [21].

Nucleic acid-based therapeutics utilize natural cellular mechanisms to induce gene silencing or protein production [127]. Nucleic acid molecules have the capacity to specifically enhance or suppress gene expression, which makes them useful for treating a number of disorders [128]. Gene silencing can be utilized in a number of ways. For instance, short hairpin RNA (shRNA)-encoding genes can be used to produce silencing molecules continuously. Thus, nuclear delivery is essential and might result in conflict with endogenous RNAi processing enzymes. On the other hand, the cytosol is the region of siRNA production, and it is there that the guide strand of the siRNA is introduced to the RNA-mediated silencing complex. Then, it associates with mRNAs to control their expression [129]. Similarly, exogenous nucleic acid must enter the cytosol, where cell translation occurs, to be translated into protein by mRNA therapy [130]. In short, the delivery mechanism is four procedures: formation of the lipoplex, binding to the cell membrane, uptake and endocytosis, and endosomal escape and release [30].

### Formation of the lipoplex

When the lipid dispersions of IDDSs are in an acidic environment, tiny unilamellar vesicles are quickly generated in the absence of nucleic acid. With acidity reducing, the neutral form is adopted by enhancing the content of ionizable lipids, which decreases intervehicle electrostatic repulsion as well as destabilizes bilateral structures produced by vesicle fusion. At the time of vesicle fusion, most auxiliary lipids (e.g., PEG-lipids, phospholipids, and cholesterol) [90] are assigned to the outer monolayer of IDDSs, and most of the ionizable lipids are partitioned to the IDDSs interior, which leads to the formation of a hydrophobic centre in IDDSs. In order to prevent further inter-IDDSs fusion, the prerequisite for reaching equilibrium is increasing PEG-lipid concentration [2].

In the existence of nucleic acid, small nucleic acid-containing vesicles are formed between two closely connected lipid monolayers. With increasing pH, the neutralization of ionizable lipids allows the fusion of various particles in empty IDDSs. The removal of PEG-lipids and phospholipids/cholesterol from the complexes is one of the factors that restrict the entire process. It needs to be mentioned that the high ethanol concentration accelerates the rate of exchange of individual lipid molecules, resulting in the quick creation of equilibrium structures. Kulkarni et al. [50] combined ionizable materials and auxiliary materials with siRNA in a pH 4 aqueous buffer by taking advantage of the positive charge properties of ionizable nanomaterials. The pH was increased to neutral or mildly alkaline after the remaining ethanol was eliminated, and then the ILNP-siRNA was finally produced. Moreover, since only a 1.5 mol % PEG-lipid-assisted liposome system is observed, it is indicated that phospholipids and cholesterol must be separated to form stable small structures on the outer monomolecular layer at pH 4. A further increase in pH results in a shift in the charge of the ionizable lipid toward neutrality, allowing for further fusion of the vesicles [50]. A prerequisite for initiating transfection is the interaction of the lipoplex with the cell membrane. The transfection process involves the binding of lipoplexes to target cell membranes, cellular uptake of lipoplexes, encapsulation of lipoplexes in endosomes, disruption of endosomes, and escape of nucleic acids.

### Binding to the cell membrane

The surface charge of ILNPs is responsible for interacting with cell membranes and the biological environment. Because the cell membrane is negatively charged, ILNPs repel with the cell membrane and are not taken up by the cell. Besides, positively charged ILNPs may directly damage the cell membrane, leading to cytotoxicity. This is why ionizable lipids are crucial in ILNP design. The ILNPs initially containing ionizable lipids are electrically neutral, avoiding any unwanted electrostatic interactions. However, it acquires a positive charge at acidic pH in the endosome. The positively charged ionizable amine groups are the main groups of ionizable lipids. IDDSs are capable of interacting with anionic mRNA and fostering membrane binding in acidic environments [115, 131]. A new class of IDDSs called iLP181 has been created by Li et al. [115]. The IDDSs were protonated in an acidic environment, had a positive charge, and interacted with the inner membrane’s anion to facilitate membrane fusing. The interaction between ILNP and cell membrane can achieve. Likewise, one of the theories of membrane instability proposes that electrostatic interactions between ionizable lipids and phospholipids in endosomal membranes lead to membrane rupture due to exposure of ionizable lipids to the acidic pH environment of late endosomes. Phospholipids show similarities to biological membranes, which may make the membranes fuse easily. Therefore, increasing the content of phospholipid can achieve the efficacy of delivery [132]. Liu et al. [24] also carried out a similar series of experiments, demonstrating that small hermaphroditic heads, as well as large tails, established more efficient fusion membranes and phase transitions from lamellar to hexagonal (H_II_).

### Uptake and endocytosis

After binding to the cell membrane, ILNPs need to enter the cell through uptake and endocytosis. A critical step in the delivery of nucleic acids into the cytoplasm is the cellular uptake of ILNPs. In particular, the cellular uptake of ILNPs is primarily dependent on endocytosis. More in detail, the cellular internalization of ILNPs is mediated by specific serum proteins on their surfaces. ILNPs with a size of about 100 megadaltons do not have the ability to cross cell membranes by passive diffusion, so they enter cells by endocytosis. Uptake and endocytosis are overwhelmingly actuated by clathrin-mediated endocytosis and micropinocytosis [133]. The amine head group structure of ILNPs is considered to be the major aspect affecting the entry of nucleic acid drugs into cells. This is because the positive surface charge of the amine head group can be protonated. The electrostatic interactions between ILNPs and the negative surface of the endosomal lumen are further promoted, which can accelerate the fusion of ILNPs with the cell membrane. The cell uptake can be enhanced and the completion of the uptake process facilitated [134]. Specifically, The IDDSs based on DODMA to deliver siRNA in cells are widely studied. Due to the tertiary amine and unsaturated hydrophobic chain, DODMA showed great fusion properties. More importantly, DODMA, carrying positive charges in an acidic environment, improved the fusion between IDDSs-siRNA and the cell membrane. Furthermore, siRNA delivery based on this system exhibited enhanced cellular uptake. The results showed that cyclin-dependent kinase 4 siRNA delivery via IDDSs may be a promising cancer therapy [33]. Endocytosis represents a mechanism for the delivery of various nanocarriers (e.g., complexes and ligand complexes) into cells. The mechanism comprises numerous entry approaches, which contain endocytosis about clathrinid and caveolae-mediated, micropinocytosis and separate entrances for caveolae and clathrin [83].

It has been revealed that the cellular uptake of ILNPs is primarily dependent on endocytosis [81]. In particular, the cellular internalization of ILNPs is mediated by specific serum proteins on their surfaces [88]. For liver-targeted ILNPs, ApoE absorbed on their surface can interact with low-density lipoprotein receptors (LDLRs) on hepatocytes [135]. The effects of clathrin-mediated (chlorpromazine) and pituitary-mediated (filipin III) inhibitors of endocytosis on intussuscept ApoE/YSK-MEND were studied. Tamaru et al. [88] found that the cellular uptake of ApoE/YSK-MEND treated with chlorpromazine was reduced by 36.9% compared to the control. Numerous experiments evaluated the quantitative relationship between ionizable lipid and ApoE derivatives, and found a dose-dependent manner rose in the cellular uptake of IDDSs [91]. Besides, as MBEC4 cells were treated with filipin III, an equivalent inhibitory effect was observed [88].

In a word, ApoE/YSK-MEND was internalized via endocytosis mediated by caveolae and chrathrin and was able to prevent lysosomal degradation. Endosomes, composed of lipid bilayer barriers, prevent foreign nucleic acids from entering cells. The endoplasmic lipid bilayer has also been an important barrier to genetic drug delivery for over 50 years. The reason why ILNPs are the preferred gene delivery vehicle is that their neutral surface charge effectively reduces toxicity at physiological pH and ultimately improves safety.

### Endosomal escape and release

The intracellular dynamics of ILNPs is an important factor affecting the efficiency of nucleic acid delivery. In particular, endosomal escape is thought to be necessary to avoid nuclear acid degradation and its efficient transfection. Endosomal escape is a major challenge for gene delivery. Some studies have shown that most nanoparticles are difficult to be released into the cytoplasm after entering endolysosomes [136–138]. ILNPs play an important role in delivering nucleic acid drugs to cells [134]. During cellular uptake, the formation of cellular vesicles (early endosomes) with a pH between 5.5 and 6.2 is required to engulf ILNPs. As early endosomes become late endosomes, the pH within them decreases to 5.0–5.5. Ultimately, fusion with lysosomes lowers the pH of the vesicles to 4.5 – 5.5. Lysosomes contain a series of enzymes capable of dismantling ILNP structures and degrading nucleic acid molecules [139]. Therefore, the majority of nucleic acid molecules should escape the endosome before degradation begins. Ionizable lipids that can adjust their charge according to ambient pH are considered to be an important part of ILNPs for endosomal escape. During endosomal maturation, ionizable lipids have cationic on their amine heads that can bind to negative lipids on endosomal membranes. This binding induces the formation of hexagonal (H_II_) structures and disruption of endosomes, leading to escape of nucleic acids [21].

The degree of bilayer disruption was found to correlate with increased percentage of ionizable lipids and cholesterol-conjugated lipids, increased aliphatic chain length (C14-C18), and longer tails with a cis double bond [95]. In particular, the properties of the lipids that make up ILNPs affect the dissociation of nucleic acid molecules from ionizable lipids. It is worth noting that chain saturation is not conducive to membrane fusion. Pegylated lipids and fusogenic lipids (1,2-dioleoyl-sn-glycero-3-phos-phoethanolamine, cholesterol) in ILNPs enable them to circulate for a long time in vivo, fuse with biological membranes, and be internalized by target cells [140]. However, it is usually the PEG-lipid analogs rather than PEG that dissociate from the complexes, which effectively prevents steric interference caused by membrane interactions [141].

The development of more efficient ionizable lipids and pH-responsive polymers or the use of endosomal escape enhancers could further enhance the efficiency of endosomal escape. Habrant et al. [30] found that the improved delivery properties of lead molecules 30 with diester linkers and two oleyl chains (hydrophobic moieties) resulted from optimized endosomal escape. They demonstrated that the ester bond in 30 has better hydrophobic interactions with negatively charged phospholipids. Moreover, the citronellal had a special structure that can successfully transfer mRNA as a membrane breaker. Citronellal-based delivery system showed the highest lipid fusion ability. The various unsaturated thiols were synthesized and combined to form 91 ionizable nanomaterials. After the experiment, it was found that the structures of ionizable nanomaterials had different functions. In particular, alkyl thiol tail was a distinctive tail structure that might aid endosomal escape [121]. Especially, the structure of ApoE/YSK-MEND not only enables efficient uptake, but also has a better endosomal escape and release. The majority of ApoE/YSK-MEND did not co-localize with lysosome markers, indicating that it may be able to effectively evade lysosomal destruction [142].

The derivatives of ionizable lipids were apparently uncharged at physiological pH, while positively charged in acidic environments and negatively charged in alkaline conditions. For instance, ILNP consisting of dioleoylglycerophosphate diethylenediamine conjugate, is nearly neutral in a neutral environment and positively charged in an acidic environment (pH 6.0) [91]. The positively charged environment-like interior of the endosome causes the endosomal membrane to rupture at low pH, resulting in the release of siRNA into the target cell cytosol. Cationic lipids interact with cell membranes depending on pH, which helps reduce their toxicity during systemic circulation. However, higher fusion rates indicate stronger interactions of ionizable lipids with endosomal membranes and endosomal escape can be further enhanced in acidic environments [105].

It is important to clarify the mechanism of endosomal escape of ionizable nanoparticles. The adsorption of protein corona to nanoparticles in vivo and how it affects nanoparticle performance is still not well characterized. After intravenous administration, how to target tissues other than liver cells? How can gene expression be turned on or off so that only specific regions of tissue are transfected? How can it be delivered to important target tissues such as the brain? Similar questions abound. Even so, optimism is needed for delivery systems based on ionizable materials to meet clinical needs. Tuning the outer surface of ionizable nanoparticles enables different tissue distributions. Co-encapsulation of drugs sensitive to external radiation can achieve precise treatment of specific sites. Optimizing the composition of increasingly functional ionizable nanoparticles will enable effective control of difficult-to-transfect tissues.

Intravenous ILNPs have been reported to bind to ApoE in serum. It is worth noting that ApoE has high affinity for LDLRs. Therefore, systemically delivered ILNPs are mainly distributed in the liver. A study has shown that the hepatic uptake of non-cationic ILNPs is mediated by ApoE, demonstrating that ILNP charge has a strong effect on their tissue distribution [135]. To improve the delivery of nucleic acid molecules in non-liver tissues, CRISPR/Cas technology was used to develop various lipid classes. Researchers have proposed that manipulating the charge of prepared ILNPs is key to achieving organ-specific delivery [143]. In addition to standard ILNP components, the authors suggested adding selective organ targeting (SORT) molecules to achieve lung- or spleen-specific gene delivery. The addition of the cationic lipid 1,2-dioleoyl-3-trimethylammonium-propane lipid (DOTAP) to ILNPs has been reported to change their tissue tropism from liver to lung [144]. And combining SORT molecules into several classes of ILNPs yielded similar results. In addition, the particle size and circulation life of ILNPs also have a great impact on improving their targeting to non-liver tissues [145]. ILNP-siRNA formulations containing PEG-DSG (persistent PEG-lipid) with a particle size of 30 nm and long circulation life in vivo have been developed in the LNCaP model for the treatment of prostate cancer [146]. However, in the central nervous system, local administration is required because ILNP-siRNA formulations cannot cross the blood–brain barrier [147]. Due to the small size of siRNA molecules, most ILNP-siRNA systems were developed. In the future, more ILNP systems need to be designed so that macromolecules can be efficiently carried.

## Applications of IDDSs in the treatment of the diseases

**Fig. 4:**
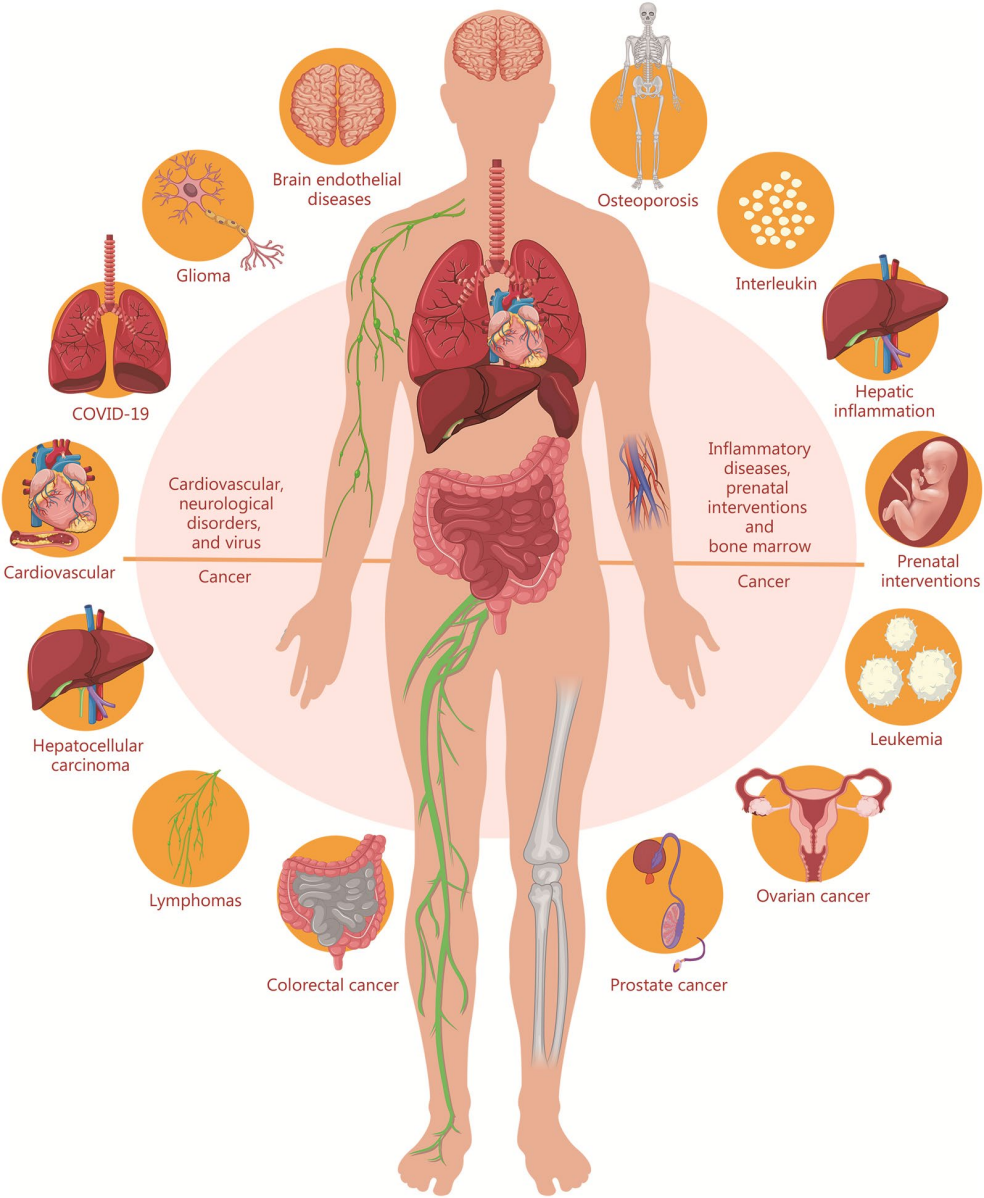
Types of diseases that can be treated by ionizable drug delivery systems. COVID-19 coronavirus disease 2019

Gene therapy has a bright prospect in inflammatory diseases, virus therapy, tumor immunotherapy, neurological disorders, prenatal interventions, bone marrow and hyperlipidemia (Fig. 4), including protein replacement and cellular genetic engineering [88]. In particular, there are more researches on the applications of IDDSs, and some products have been produced and put into the market (Table 2). Onpattro^®^ is the first IDDSs approved by FDA in 2018, and Spikevax^®^ and Comirnaty^®^ were subsequently approved in 2021. LUNAR^®^, a liposomal platform consisting of a specific ionizable amino lipid ATX, has been developed by Arcturus Therapeutics, Inc. (SanDiego, CA, USA) for RNA delivery and has shown promising results. LUNAR^®^ in preclinical and clinical trials mainly aimed at ornithine transcarbamylase deficiency, cysticfibrosis, influenza, hemophiliaB, as well as the COVID-19 vaccine. In addition, the products CvnCoV, LNP-nCoVsaRNA and ChulaCov19 are made up of ALC-0315, A9, CL1 for the COVID-19 vaccine, respectively. ARCoV and PTX-COVID19B are also undergoing clinical trials. Moreover, NTLA-2001 is used for the treatment of ATTR, which is used in ongoing clinical trials.

**Table 2:** Clinical products produced using ionizable nanocarriers

Ionizable nanomaterials	Nucleic acid	Application	Product	Clinical Phase
SM-102	mRNA	COVID-19 vaccine	Spikevax^®^	Approved in 2021
Acuitas ALC-0315	mRNA	COVID-19 vaccine	Comirnaty^®^	Approved in 2021
MC3	siRNA	ATTR	Onpattro^®^	Approved in 2018
ALC-0315	mRNA	COVID-19 vaccine	CVnCoV	III
A9	mRNA	COVID-19 vaccine	LNP-nCo VsaRNA	I
CL1	mRNA	COVID-19 vaccine	ChulaCov19	I
ATX	mRNA	COVID-19 vaccine	LUNAR-COV19 (ARCT-021)	III
-	mRNA	COVID-19 vaccine	ARCoV	III
		COVID-19 vaccine	PTX-COVID19B	II
ATX	mRNA	Ornithine transcarbamylase deficiency	LUNAR-OTC (ARCT-810)	II
		Cystic fibrosis	LUNAR-CF (ARCT-032)	Preclinical
		Influenza	LUNAR-FLU	Preclinical
		Hemophilia B	LUNAR^®^	Preclinical
LPO1	sgRNA	ATTR	NTLA-2001	I

*SM-102* Heptadecan-9-yl 8-[(2-hydroxyethyl)(6-oxo-6-(undecyloxy) hexyl) amino] octanoate, *MC3* (6Z,9Z,28Z,31Z)heptatriaconta-6,9,28,31-tetraen-19-yl-4-(dimethylamino)butanoate, *ALC-0315* 6-[(2-hexyldecanoyl)oxy]-N-{6-[(2-hexyldecanoyl)oxy]hexyl}-N-(4-hydroxybutyl)hexan-1-aminium, COVID-19 coronavirus disease 2019, ATTR transthyretinamyloidosis, *mRNA* messenger RNA, *sgRNA* small guide RNA

### Inflammatory diseases

It has been reported that IDDSs targeting immune cells are promising for siRNA therapy of inflammation. The application of IDDSs composed of various ionizable nanomaterials in the delivery of siRNA to immune cells has been extensively studied. For example, IDDS-based gene-silencing effects of target protein on macrophages have been successful in vivo, and this system is expected to be extended to various immune diseases via siRNA delivery [44]. In a recent study, Ding et al. [148] designed IDDSs with spermine as head to form top performance IDDSs (114-LNP), which was used to deliver siRNA for silencing fundamental pro-inflammatory cytokines such as interleukin-1β (IL-1β) in macrophages. The IDDSs accomplished available endosomal escape in macrophages and internalized efficiently siRNA via multiple endocytosis pathways. IDDSs/siIL-1β can efficiently control IL-1β expression to achieve the purpose of attenuating hepatic inflammation and liver damage in an acute liver failure model [149].

In particular, Hepatitis B is very contagious [150] and is highly prevalent in Asia and Africa [151]. The disease will aggrandize the possibility of cirrhosis or liver cancer [152], requiring prevention and treatment [153] to meet the demand of ILNP [154]. ILNP could bind to ApoE, and the protein conjugate could cross the hepatocyte membrane through LDLR-mediated endocytosis. However, LC8-based ILNP-RBP131 never bound to ApoE and was not mediated by LDLR. It was speculated that RBP131-siRNA was mainly concentrated and stored in hepatocytes through apertured sinusoidal capillaries [155]. In addition to ionizable lipid-mediated drug delivery systems, ionizable polymeric nanosystems have also been developed for the treatment of inflammatory diseases. As we all know, TNF-α secreted by macrophages, as a pro-inflammatory cytokine, can also cause a series of inflammatory problems including hepatic inflammation. Furthermore, mannose-modified trimethyl chitosan-cysteine-siRNA nanoparticles effectively protected rats from acute liver injury by reducing TNF-α mRNA levels in a range of tissues including the liver [156].

### Virus infection

In 2016, Swaminathan et al. [157] published their major research about a tetravalent sub-unit Dengue vaccine. They described the formulation of the vaccine including highly well-tolerated and effective ionizable cationic lipids. The safety and efficacy of IDDS vaccines need to be further monitored in response to the emergence of dengue virus and other new viruses. The severe COVID-19 caused grievous harm in economy, life, health, and medical treatment nowadays [158, 159]. Data on COVID-19 has been systematically studied and analysed by researchers [160].

In particular, as shown in Table 2, IDDS-based COVID-19 vaccines have been widely developed and some have entered the clinical stage [161]. For example, in a study, following rapid distribution of the Pfizer-BioNTech vaccine containing ALC-0315 and the Moderna vaccine containing SM-102 under an EUA by the FDA, survey results among nursing home residents indicated vaccine effectiveness ranging from 53 to 92% against SARS-CoV-2 infection [111, 162]. The efficacy of the Pfizer-BioNTech vaccine and the Moderna vaccine was similar, however, the vaccine was less effective after the Delta variant became popular in the United States. During the Delta period, the adjusted vaccine effectiveness was only 53.1% in an analysis of 85,593 reports from 14,917 facilities. Additionally, the S-2P mRNA-LNP vaccine and the Protein-3 M-052-SE vaccine were highly immunogenic in infant rhesus macaques, setting the stage for the development of a SARS-CoV-2 vaccine for infants [163]. Moreover, nanovaccines were created to treat a number of disorders. Ionizable polymeric nanomaterials have also drawn extensive attention as vaccine platforms due to their synthetic feasibility, low immunogenicity, and high biodegradability.

### Cancers

The essence of cancer is a genetic mutation that causes cells to grow out of control [164]. If left untreated, malignant tumors can invade and attack neighbours or even distant organizations. Radiation [165] and chemotherapy [166] have limited efficacy and significant side effects. The tumor place, fearful toxicity, genetic incompatibility, or other factors result in poor response rates. The secret to successful cancer treatment is taking advantage of specific pathogenic mechanisms and avoiding oncogene mutations that lead to cellular functions and biological pathways [167]. Nearly all cancers have a unique genetic code responsible for the mechanism, making traditional cancer treatments more difficult. Due to heterogeneity, cancers need to be treated as distinct therapies even if they share the same diagnosis/classification [102].

The encapsulation of tumor antigens encoding nucleic acids by IDDSs had a dual role, both preventive and therapeutic [123]. It has been reported that ionizable lipids can deliver nucleic acid drugs to hepatocytes through an active liver-targeting mechanism. For example, iLP171 was a unique ionizable lipid to access mRNA transfection efficiency and protein expression in hepatocytes. iLP171 could efficiently wrap mRNA to form regular spherical structures and be taken up by Huh7 cells, indicating that mRNA was efficiently transported to the liver. The highest expression of mRNA was observed about 6 h after transfection into Huh7 cells with no obvious adverse reactions. Since current studies on mRNA delivery were scarce in liver-related diseases (e.g., anemia and hepatocellular carcinoma), the studies on IDDSs for mRNA provided new research directions [168]. The ionizable lipid nanoparticle (iLP181) encapsulating psgPLK1 had also been used to study the treatment of hepatocellular carcinoma. The psgPLK1 was the optimal plasmid for expressing Cas9 protein and sgRNA targeting PLK1, to examine its ability to deliver a CRISPR/Cas system. The PLK1 gene was successfully edited in HepG2-Luc cells using the iLP181/psgPLK1 nanoformulation. The accumulation of iLP181/psgPLK1 in tumors was observed for at least 5 d after intravenous injection. IDDSs can be delivered to the whole body, targeting not only the liver but also the spleen [115]. Using the [^3^H]-labeled nucleic acid [^3^H]-SSB, Christensen et al. [169] exploited the in vivo delivery of DLin-KC2-DMA. The drug was transported to the whole body with high concentration in blood, such as the spleen, liver and stomach. After seven days of administration, the concentration in blood decreased significantly. The main organs of radioactivity, according to imaging, are the spleen and/or the liver. When administered systemically, ILNPs may bind to ApoE in the circulation, then interact with LDLR on hepatocytes, and finally be internalized by hepatocytes through LDLR-mediated endocytosis [135, 170].

Improper regulation of B lymphocyte proliferation may result in at least 50,000 new cases of non-Hodgkin B-cell lymphoma each year in China. However, current mRNA delivery systems mainly transfect hepatocytes but cannot target B lymphocytes. Fenton draws our attention to lymphomas by synthesizing ILNPs targeting B lymphocytes. B cell dysfunction may be at the root of disease and plays a crucial part in disease prevention and management [171]. Fortunately, the development of ILNPs has opened up a novel avenue for the treatment of lymphoma. In particular, OF-Deg-Lin containing degradable and ionizable lipids was synthesized by a three-step method [171]. And OF-Deg-Lin-mRNAs induced more than 85% protein generation in the spleen containing abundant B lymphocytes, although they were transiently observed in other organs. Moreover, chimeric antigen receptor T-cell therapy has also been demonstrated for the treatment of lymphoma through mRNA delivery. Billingsley et al. [90] synthesized a ILNP library by combining 24 different ionizable lipids with auxiliary lipids and utilized different ILNPs to deliver luciferase mRNA to primary human T cells (Jurkat cells). They found that the top formulation C14-4 ILNP induced chimeric antigen receptor expression levels comparable to electroporation. This indicated that ILNP was able to deliver mRNA to T lymphocytes to induce protein expression.

Selective drug delivery with cationic nanoparticles for the treatment of gliomas is considered a viable strategy due to the abundant presence of anionic lipids on the surface of glioma cells. Moreover, tumor selectivity can be further enhanced by protonation of ionizable carriers in the acidic environment outside glioma cells. Sonodynamic therapy [172] and radiation therapy [172] for glioblastoma have been studied extensively in the past. Nowadays, IDDSs are expected to provide better therapeutic results. The ionizable cation lipids enhanced deposition and penetrated into the glioma core, while non-ionizable micelles appeared bad potency by semiquantitative fluorescence analysis [173]. Cationizable lipid micelles as drug delivery vehicles can be used for selective treatment of gliomas by intra-arterial injection.

Due to its aberrant expression in prostate cells, protein kinase N3 (PKN3) has been developed as a target for nucleic acid (e.g., shRNA and siRNA) therapy. The creation of obstructing sequences and delivery mechanisms is a significant current problem in PKN3 RNAi treatment. Wang et al. [174] synthesized a multifunctional ILNP (DDA-SS-DMA) that integrated ester bonds and disulfide bonds for PKN3 shRNA delivery and prostate cancer therapy. In particular, shPKN3-3357 and shPKN3-2459 were also developed as novel PKN3 shRNA sequences. The results showed that the ILNP-shPKN3-2459 treatment group produced a high tumor inhibition rate (65.8%) and lower toxicity in vivo. Besides, siRNA ILNP formulations for silencing the androgen receptor in prostate cancer have also been developed. The effect of siRNA ILNP formulations also silenced AR in prostate cancer cell lines and in LNCaP xenograft tumors. The results showed that ILNP containing the ionizable cationic lipid (DLin-KC2-DMA) effectively silenced the AR gene in both wild-type AR-expressing cells (LAPC-4) and LNCaP xenograft tumors. This study also suggested that strategies to improve targeting of ILNP AR-siRNA systems to prostate cancer cells may be more effective in knocking down AR and treating advanced prostate cancer [174, 175].

In epithelial ovarian cancer, tumor cells typically aggregate into spheroids. These spheroids have a core composed of mostly cancer stem-like cells that control tumor growth and recurrence. If the core could be approached, nanomedicines may reach their greatest potential to reduce tumor recurrence rates. Therefore, the researchers developed various strategies to maximize the penetration of the payload into the stem-like cells. One of the delivery systems with great potential is submicron ILNP dispersions of ionizable lipids. These ionizable lipids improve circulation time and deliver nucleic acid drugs intact to target cells. Tal et al. [176] mimicked the tumor microenvironment of ovarian cancer by culturing 3D spheroids and developed an ILNP-based approach to penetrate the tumor core. They found that MC3-containing ILNPs could efficiently penetrate spheroid cores made of NAR (NCI/ADR-Res) human ovarian cancer cells. They also suggested that the gene knockdown effect of this system in spheroids of epithelial ovarian cancer should be further investigated. However, there are few reports on the application of IDDSs in colorectal cancer therapy [176]. Since cancer cells clear high-density lipoprotein (HDL) particles by expressing scavenger receptor type B1, Shahzad et al.[177] integrated siRNA into recombinant HDL nanoparticles (rHDL) and efficiently delivered siRNA to human epithelial ovarian cancer cells and human colorectal cancer cells via a scavenger receptor type B1-mediated pathway. They found that these nanoparticles effectively silenced the expression of proteins (FAK and STAT3) critical for the growth and metastasis of ovarian and colorectal cancers. In particular, FAK siRNA/rHDL therapy selectively inhibited the growth and metastasis of colorectal and ovarian cancer cells without affecting other organs. In conclusion, the development of rHDL nanoparticles opens new horizons for gene therapy of malignant tumors.

Current treatments do not take into account the heterogeneity of leukemia patients and may cause side effects. Therefore, new treatments need to be investigated to target the disease through the patient’s molecular fingerprint. The emergence of gene therapy opens new avenues for the treatment of leukemia. Chromosomal translocations are thought to be driver mutations in leukemogenesis. Researchers speculated that fusion oncogenes in the hematopoietic system could serve as therapeutic targets. The ILNP-siRNA formulation containing DLin-MC3-DMA lipids targeted the fusion oncogene BCR-ABL in chronic myeloid leukemia cells. siRNA could be efficiently delivered by ILNP formulations in vivo and ILNP-siRNA were almost 100% taken up by bone marrow of leukemia models. In particular, mice treated with ILNP-BCR-ABL siRNA had less disease burden compared to ILNP-CTRL siRNA. This study demonstrated that fusion oncogenes could be used as specific targets for gene therapy to achieve personalized treatment of leukemia patients [178].

### Neurological diseases

Although numerous IDDSs have been designed to deliver payloads into target tissues, ionizable nanocarriers targeting brain tissue remain to be investigated. Positively charged peptides and receptor-specific ligands are known to increase the transport of nanocarriers across the blood–brain barrier (BBB). RNA aptamers have been shown to be useful in the design of ionizable nanocarriers with greater specificity for antigen-expressing cells [88, 179, 180]. For example, Ray et al. [180] found that DLin-MC3-DMA-containing ILNPs loaded with a CC chemokine receptor type 5-selective RNA aptamer could bypass BBB penetration and be efficiently taken up by CC chemokine receptor type 5-expressing cells. They also showed that ILNPs with cell penetrating peptides did not significantly promote ILNP uptake by cells through the BBB. ILNP preparations have been shown to be safe, cross the BBB, and be taken up by various cells. Brain endothelial cells make up a significant portion of the BBB. The neurovascular unit containing numerous brain endothelial cells is of great importance for the maintenance of normal brain function. Furthermore, Tamaru et al. [88] synthesized an ApoE-modified ionizable nanocarrier ApoE/YSK-MEND and investigated its ability to target brain-derived endothelial cells. The results showed that the gene silencing effect of the nanocarrier was positively correlated with the content of ApoE. The ApoE-modified YSK-MEND loaded with plasmid DNA also produced higher gene expression in mouse brain ventricles compared with unmodified YSK-MEND. The nanocarriers may be useful for gene delivery in neural progenitor cells and the treatment of neurodegenerative diseases.

### Other diseases

In addition to inflammatory diseases, viral infections, cancers, and neurological diseases, IDDSs have also been developed for prenatal interventions, bone diseases, and cardiovascular diseases.

#### Prenatal interventions

Prenatal gene therapy can treat congenital diseases at an early stage to reduce morbidity and mortality. It can treat and expand larger numbers of progenitor cell populations than postnatal therapy. DLin-MC3-DMA achieved mRNA delivery to fetal liver more efficiently via intra-amniotic injection. Results demonstrated that mRNA delivered to the liver successfully induced the generation of therapeutic secreted proteins [181, 182]. Briefly, prenatal gene therapy offers fresh perspectives on the in-utero delivery of nucleic acid for protein replacement therapy and gene therapy. Ionizable nanomedicine is opening the way for some highly attractive research avenues in prenatal interventions, creating opportunities for personalized treatment.

#### Bone diseases

Lineages of osteoblast cells have been reported in bone marrow mesenchymal stem cells (BM-MSCs) as a regenerative medicine tool for the treatment of various genetic diseases. MSCs have broad differentiation and proliferation capabilities and can be amplified by genetic engineering tools to express therapeutic proteins. Nonetheless, the development of gene delivery systems for targeting MSCs remains a challenge. Various materials for the treatment of bone injuries have been systematically studied [165]. For osteoporosis, the application of pDNA to deliver various therapeutic genes is only in its infancy. Since BM-MSCs are difficult to transfect and reach due to their biased location in the bone marrow, intravenous administration is often required to deliver nucleic acid drugs to patients with osteoporosis. According to the characteristics of osteoporosis, ionizable nanocarriers with appropriate size and surface properties should be designed so that they can stay in the blood longer and accumulate efficiently in the bone marrow. Vhora et al. [183] developed a novel ILNP preparation with an ionizable head group (Boc-His-ODA/BHODA and His-ODA/HODA) and a hydrophobic C18 tail to deliver the pDNA encoding bone morphogenetic protein-9 (BMP-9) to BM-MSCs for osteoinduction. They found that the ILNP formulation had smaller size, serum stability and low hemolytic capacity, indicating its suitability for intravenous injection. In vitro results showed that the prepared ILNP preparations exhibited weak cytotoxicity, non-significant induction of reactive oxygen species, and high transfection rates. The in vivo results indicated that the ILNP formulation exhibited better performance in terms of safety and bone regeneration in OVX rats. The results of high-resolution X-ray showed that the radiopacity of the femur and lumbar vertebrae of the rats in the group of two ILNPs (HODA-LNP and bone-homing peptide targeting HODA-LNPT) loaded with the BMP-9 gene was significantly increased in comparison with that of the control group. Compared with the HODA-LNP group, the effect of the HODA-LNPT group was higher. MicroRNA-loaded polysaccharide nanoparticles have been reported to enhance chondrogenesis. The Phe-conjugated γ-PGA NPs (γγ-PGA–Phe NPs) were more effectively absorbed by bone marrow-derived immature dendritic cells [184]. The proteins were transported to bone marrow cells through endocytosis for all kinds of bone diseases.

#### Angiocardiopathy

The main cause of angiocardiopathy is vascular blockage, which is mainly caused by arteriosclerosis. The process of angiocardiopathy starts from normal blood vessels, which may lead to coronary atherosclerosis due to a large amount of fat or other reasons, and gradually evolves into vascular stenosis and even vascular occlusion. Abnormal lipid metabolism in the liver can lead to hyperlipidemia and lipids in the blood adhere to the walls of blood vessels. Hyperlipidemia may lead to various genetic [185] and acquired disorders, especially severe cardiovascular disease [186, 187]. Hu et al. [188] developed an ionizable lipid assisted nucleic acid delivery system (iLAND) based lead lipid (A1-D1-5) for the delivery of siRNA targeting angiopoietin-like 3 or apolipoprotein C3. iLAND was found to be highly safe and low-dose siRNA effectively reduced serum cholesterol and triglycerides in high-fat diet-treated mice and human apolipoprotein C3 transgenic mice and db/db mice. This study showed that IDDSs are of great significance in the treatment of hyperlipidemia and prevention of cardiovascular disease through gene therapy. Hyperlipidemia is easy to form thrombus, and thrombus is also an important cause of cardiovascular disease. The optimized poly(lysine)heparin electrostatic assembly nanoparticles to modify the surface can effectively block the coagulation pathway and reduce thrombosis [189]. Novel ILNPs loaded with siRNA have also been developed for hyperlipidemia [188].

IDDSs are accelerating a revolution in medicine. Almost any disease imaginable can be treated with gene therapy. In addition, these drugs can be developed at an astonishing rate. As long as the protein to be silenced or expressed is known, the required nucleic acid (e.g., siRNA and mRNA) can be synthesized within 1–2 months, and packaged into nanoparticles within 1–2 d to form a targeted drug. The paradigm of rapid drug advancement from concept to clinical application of a COVID-19 mRNA vaccine after 3 months of viral genome sequencing demonstrates these possibilities. A bigger potential is to use the liver as a bioreactor to produce whatever protein is needed after intravenous injection. Its applications range from monoclonal antibodies to treating cancers, infections and other diseases.

## Future perspectives and challenges

**Fig. 5:**
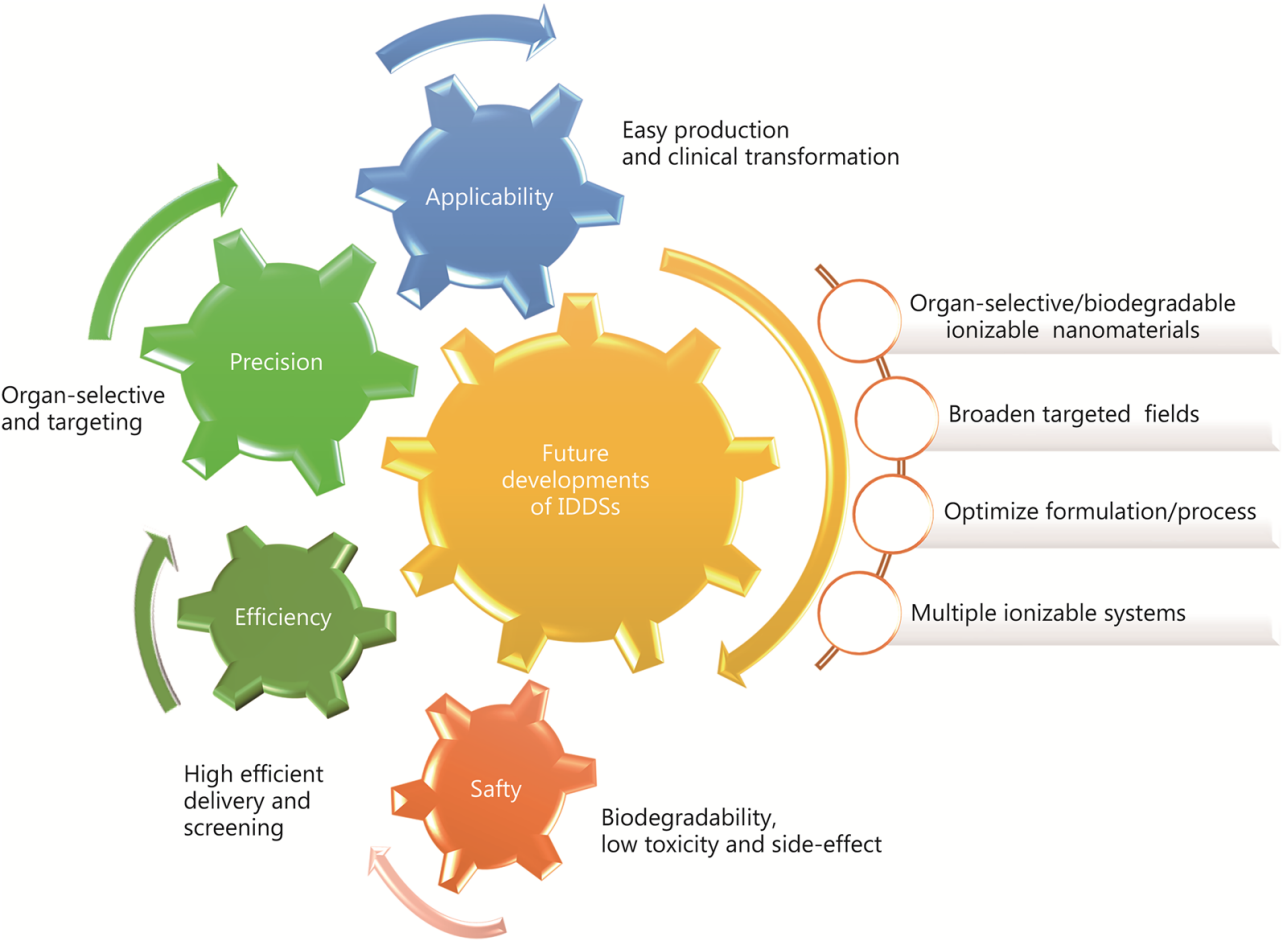
Future developments of ionizable drug delivery systems. IDDSs ionizable drug delivery systems

Although the IDDSs have witnessed incredible growth, there are still various challenges in clinical transmission and efficacy (Fig. 5). For the future design of new IDDSs, we propose a safty-efficiency-precision-applicability rule that includes the design of biodegradable IDDSs with low toxicity and side-effect (Safty), efficient drug encapsulation and delivery as well as high-throughput screening compatibility (Efficiency), good organ-selectivity (Precision), and easy production process and clinical transformation (Applicability). The ultimate objective of precise and selective application of IDDSs in clinic will be determined by the multiple complicated factors as above mentioned. Researchers need to take into consideration of the defects and disadvantages to design and develop the next-generation gene vectors.

### Safety rule

The rule of safety is indicated as biodegradability, low toxicity and side-effects. In the three types of IDDS, the ionizable polymeric nanosystems and ionizable polymer-lipid delivery systems contain biodegradable polymers showing high natural superiority of in vivo biodegradability. However, the influence of polymers on cell membrane proteins can’t be ignored. Therefore, rational design of ionizable polymers and polymer-lipids with decreasing cell membrane toxicity is important to achieve a highly safe profile of IDDSs.

Researchers have conducted extensive researches on ionizable polymeric nanosystems, among which, polysaccharide nanoparticles, especially chitosan nanoparticles, were generated for the transport of nucleic acids. They have the advantages of high positive charge density, low cost, low cytotoxicity, good biodegradability, and low immunogenicity. But their transfection efficiency varies depending on the molecular weight and degree of deacetylation of chitosan, nucleic acid concentration, N/P ratio, charge ratio, serum concentration, pH value of the medium, etc.. Through different routes of administration, chitosan can be used to address the problem of poor cellular uptake of naked siRNA in vivo. The research of these aspects in animal models needs to be further expanded for clinical trials. Poly(lysine)/heparin has the advantage of protecting cationic polymers from serum interference, and the disadvantage of heparin itself can not be used as a gene carrier. Research on the interaction between cationic polymers and nucleic acids is currently focusing on electrostatic interactions, while the effects of molecular structure and dynamics remain to be clarified. The heparin concentration and dissociation time required for complex dissociation may be related to the type of nucleic acid and cell, which needs to be further studied to lay the foundation for its clinical application. In addition, nanoparticles based on amphiphilic polypeptide/poly(amino acid) complexes have the advantages of good biocompatibility and biodegradability since the amino acid residues can be infinitely combined and modified. However, the properties of peptide-based hybrid nanocarriers are not only affected by the peptides but also by the lipids, polymers or inorganic components. Laboratory synthesis of such advanced peptide-based nanomaterials often requires multiple steps and intensive labour work.

For ILNPs, developing more biodegradable ionizable lipids will be an important task to enable fast elimination of lipid nanoparticles from plasma and tissues, improving their safety and tolerability. For example, the clinically approved ILNPs of BNT162b2 vaccine against COVID-19 indicated the successful use of biodegradable ionizable lipids.

### Efficiency rule

The rule of efficiency for the design of IDDSs is indicated by the efficiency of drug encapsulation and delivery, as well as the compatibility of high-throughput screening.

The design of IDDSs is challenging and would be a long journey despite the recent progress made in the research process. To expedite the process of developing more applicable products, some advanced techniques, especially computer-aided formulation design and artificial intelligence-driven formulation design, show good potential for advancing the high-throughput screening of ionizable nanomaterials and nanocarriers.

Based on the ionizable strategies of lipids, the ionization optimization may be applied to other excipients to enhance the property of the whole system. Recently, the ionizable phospholipid has been reported [24]. In the future, the design of ionizable cholesterol and the combination of such ionisable cholesterol with ionizable lipids and ionizable phospholipids into one composite pluralistic carrier can be a promising direction.

### Precision rule

To achieve precise gene therapy, organ selective IDDSs are required for the targeted delivery of nucleic acid medicines to particular tissues. The organ-selectivity of IDDS can be achieved by designing ionizable organ-selective nanomaterials and targeting modification of IDDS.

Organ selectivity can be achieved by changing the internal and/or external charge of the IDDS. As reported, several IDDSs have been devoted to selectively site-specific targeted delivery in the lung, spleen, and liver. After reaching the right proportion of positive charge, targeted delivery to the liver can be achieved. The positive charge ratio is increased to allow for a convergent transfer of tissue from the liver to the lung, and increasing the negative charge of certain structures can promote spleen-specific transport [24]. Studies have shown that increasing the proportion of auxiliary materials can also improve organ selection [190, 191]. However, the organ-selectivity of currently reported IDDSs is limited; it is urgent to further explore a new generation of ionizable lipids targeting other tissues and organs such as heart, brain, etc..

### Applicability rule

The applicability rule shows mainly the easy production process and clinical transformation. To promote the successful clinical transformation, some formulation-associated considerations should be addressed, such as preparation techniques, production process, and storage stability.

The preparation techniques can not only affect the transfection efficiency by affecting the size of the nanoparticles, but also determine whether large-scale production can be achieved. Recently, the microfluidic hydrodynamic focusing and staggered herringbone mixing show greater advantages than the conventional direct mixing, ethanol injection and thin film dispersion methods, all of which are unscalable and irreproducible [192]. The advanced chip-based microfluidic devices and laminar flow rapid mixing methods are perspectives and required for rapid Good Manufacturing Practice manufacturing in the future.

The ionizable lipid-mediated delivery systems require not only ionizable lipids but also other auxiliary lipids, which makes the production process complicated. Higher requirements are necessary to develop more functional ionizable materials for reducing the industrial burden and improving production efficiency. The ionizable nanomaterials can be broadened in diversity and range, such as ionizable phospholipids and cholesterol to develop some multiple-ionizable systems. Ionizable polymer-lipid nanoparticles are easy to be fabricated due to the self-assemble formulation process, and have various modifiable possibilities. However, their endosomal release and targeting capabilities need to be further optimized to promote clinical transformation. It is also necessary to explore the influence of compositions of ionizable polymer-lipids on the endosomal escape and explain the mechanisms. Molecular self-assembly allows the development of thermodynamically stable polypeptide nanoparticles at a faster and cheaper cost, which paves the way for their clinical application.

The instability during the preparation, storage and in vivo processes is another significant limit to the clinical transformation and application of IDDSs. It was indicated that the stability could be compromised by some enhancing strategies. For instance, although increasing the amount of unsaturation in the tails of ionizable lipids can further enhance the endosomal escape, it also decreases the stability of nanoparticles. Therefore, a balanced consideration of nanoparticle stability affected by multiple factors is needed in the design of IDDSs. According to the package insert of mRNA vaccines developed by BioNTech, it has to be stored at ultralow temperature (–80 °C) and be discarded once at room temperature for less than 1 d. To solve this problem, some formulation techniques such as freeze-drying technology may be applied for next-generation products. The strategies (e.g., PEGylation, ligand modification, photochemical internalization and application of membrane fusion peptides) adopted to address these problems need to be further studied to expand their clinical applications.

## Conclusions

With the recent approval of BNT162b2 vaccine against COVID-19, IDDSs have been thoroughly recognized as the most efficient and promising nonviral system of nucleic acids for producing clinically approved products. The IDDSs play critical roles in improving the encapsulation efficiency and stability of nucleic acids, and enhancing endosomal escape to achieve functional delivery. Improving the efficacy of gene therapy depends on the rational design of new IDDSs with efficient and targeted delivery ability. In this review, we summarized the classification and characteristics of IDDSs in relation to drug delivery performance and analyzed the advantages, challenges and prospects. To promote the development of more applicable products for clinical treatment, a safety-efficiency-precision-applicability rule is proposed to design new generations of IDDSs with multiple functionalities for the progress of gene therapy.

## Data Availability

Not applicable.

## Abbreviations

ALPs: Asymmetric liposome particles
ApoE: Apolipoprotein E
BBB: Blood–brain barrier
BM-MSCs: Bone marrow mesenchymal stem cells
DODMA: 1,2-Dioleyloxy-N,N-dimethyl-3-aminopropane
DSBs: Double-strand breaks
hATTR amyloidosis: Hereditary transthyretin-mediated amyloidosis
HDL: High-density lipoprotein
IDDSs: Ionizable drug delivery systems
IL-1β: Interleukin-1β
iLAND: Ionizable lipid assisted nucleic acid delivery system
ILNPs: Ionizable lipid nanoparticles
IPLNPs: Ionizable polymer-lipid nanoparticles
LUVs: Large unilamellar vesicles
MLP: O’^1^, O^1^-[3-(dimethylamino)propane-1,2-diyl] 16-bis[2-(2methyl-5-nitro-1*H*-imidazol-1-yl)ethyl] di(hexadecanedioate) liposomes
mRNA: Messenger RNA
PBAE: Poly (β-amino ester)
PDMAEMA: Poly (dimethylaminoethyl methacrylate)
pDNA: Plasmid DNA
PEG-lipids: Polyethylene glycol-lipids
PEI: Polyethyleneimine
PGA: Poly(glycolic acid)
PKN3: Protein kinase N3
PSS: Polystyrenesulfonate
rHDL: Recombinant HDL nanoparticles
ROP: Ring-opening polymerization
saRNA: Small activating RNA
sgRNA: Small guide RNA
siRNA: Small interfering RNA
TNF: Tumor necrosis factor
